# Real-time cognitive-affective dynamics of failure feedback in a technology-based learning task

**DOI:** 10.1038/s44271-026-00487-8

**Published:** 2026-06-12

**Authors:** Helene Ackermann, Anna L. Lange, Hanna Dumont, Verena V. Hafner, Rebecca Lazarides

**Affiliations:** 1 Department of Educational Sciences, Universität Potsdam, Potsdam, Germany; 2 Science of Intelligence, Research Cluster of Excellence, Berlin, Germany; 3 Department of Computer Science, Humboldt-Universität zu Berlin, Berlin, Germany

**Keywords:** Psychology, Education

## Abstract

As technology-based learning environments increasingly employ automated feedback, understanding how learners process feedback in real time is essential. This study examined how automated cognitive and metacognitive failure feedback delivered by a humanoid robot affected performance and how effects were moderated by feedback characteristics and learner characteristics. Ninety adults (18-59 years, *M*_age_ = 29.53, 61 female, 27 male, 2 diverse) completed a learning task in three conditions: (1) fixed guidance condition with fixed-frequency and content-generic feedback, (2) basic-adaptive condition with frequency-adaptive but content-generic feedback, or (3) personalized-adaptive condition with frequency-adaptive and content-personalized feedback adjusting content to learners specific errors and prior steps. A three-level generalized path model (trials nested within time blocks within learners) was estimated to investigate effects of failure feedback on immediate task performance and cross-level moderation effects. Results showed that cognitive and metacognitive failure feedback increased the likelihood of a correct subsequent response across conditions. Relative to fixed guidance (condition 1), the implemented form of frequency-adaptive feedback (condition 2) did not show statistically significant moderation to these effects. Content-personalized feedback (condition 3) reduced effectiveness of cognitive failure feedback on immediate performance but improved overall performance as compared to content-generic feedback (condition 2). Across conditions, learners with higher cognitive ability benefited less, while those reporting higher momentary on-task boredom benefited more from cognitive feedback. These findings highlight that the effectiveness of automated failure feedback depends on both its design and learners’ situational cognitive and emotional states, illustrating how a situational, temporally sensitive approach can help open the “black box” of feedback effectiveness.

## Abstract (web summary)

This study examines how automated failure feedback influences performance in a technology-based learning task. Trial level analyses show that effectiveness depends on feedback design and learners’ situational emotional and cognitive characteristics.

## Introduction

In recent years, educational research has shown increasing interest in technology-based learning environments that provide learners with automated feedback. These environments, which include for example intelligent tutoring systems (ITS), educational games or robotic agents, are often seen as promising because they offer possibilities for providing individualized support and feedback to a large number of learners^1^. However, the evidence of their effectiveness remains mixed, as outcomes often depend on the specific ways in which the feedback is designed, implemented, and processed by learners^2^. Thus, designing automated feedback that is effective in supporting learning remains a challenge. Unlike human teachers, who can intuitively assess learners’ needs and respond to cognitive or motivational-affective cues in real time, digital systems have to rely on observable traces of learning, such as performance measures like accuracy rates or response times^3,4^. As a result, most implementations to date have focused primarily on adapting to learners’ performance and knowledge states, while more complex emotional and motivational processes are rarely considered^5^. At the same time, little is known about how students actually interpret and respond to feedback, for example, how they make sense of it and translate it into learning actions, making the learner’s perspective a “black box” in feedback research^6^. Investigating the real-time cognitive and affective dynamics of automated feedback in technology-based learning environments is therefore essential to determine how such feedback should be designed to best support learning. This study aims to contribute to that understanding.

Feedback can be broadly defined as the process in which an external agent (e.g., a teacher, peer, or parent) provides information about certain aspects of an individual’s task performance^7,8^. It has long been recognized as one of the most powerful influences on learning and achievement, although researchers still lack a complete understanding of the conditions that make it most effective^9^. A literature review shows that mostly, feedback effectiveness is assessed through self-report measures^10^. However, researchers argue that even if feedback is liked and perceived as timely and useful, it is not truly effective unless it has an objective impact upon learners’ subsequent outcomes^9^. From this perspective, it would be more valuable to focus on how learners actually behave when they receive feedback, rather than focusing primarily on their self-reported consequences^10^. This is why researchers are increasingly examining the effectiveness of feedback interventions by assessing their impact on learners’ actual objectively measured subsequent performance^6,9^. Theoretically, the effects of feedback on performance can be explained by feedback intervention theory^7^. According to this theory, feedback exerts its influence on learners’ performance by changing their attentional focus: it may direct attention to the task, to the self, or to meta-task processes (i.e., higher-level processes above the task, such as self-related evaluation)^7^. When feedback maintains attention on the task, it supports performance and learning, whereas when it shifts attention away from the task, negative effects may result. Kluger and DeNisi’s^7^ review of 131 studies demonstrated that feedback interventions overall had a positive effect on performance (*d* = 0.38). However, they also revealed that, in around one-third of the studies, performance decreased when feedback was provided. Similarly, other meta-analyses confirm that feedback in general benefits learning, but its effectiveness strongly depends on how it is designed. For instance, a review of 12 meta-analyses revealed a strong positive effect of feedback on performance (*d* = 0.73) and identified feedback as one of the most powerful factors influencing learners’ achievement^8^. A more recent meta-analysis reported a medium-high overall effect (*d* = 0.48), but again also highlighted substantial heterogeneity, with about 17% of effects being negative^11^. Thus, as feedback’s effect on performance is so inconsistent, it is important for researchers to look more closely at research findings to explain why these differences occur^6^.

One line of research on feedback effectiveness highlights the characteristics of feedback as a key factor in explaining these mixed results. Based on Panadero & Lipnevich’s^12^ systematic review of major feedback models, feedback characteristics can be classified on four levels: content (what the message says), function (its intended impact), presentation (how it is delivered), and source (who provides it). According to Narciss^13^, the design and quality of a feedback message depend especially on three facets—its content, its function, and its formal presentation—which together determine how well the feedback supports learning^14^. Feedback content can range from simple verification about correctness to more elaborated information that explains errors, provides conceptual clarification, or guides toward improvement^13^. The content of the feedback is an important moderator of feedback effectiveness. Empirical findings reveal, for example, that the more information about the task the feedback provides, the more effective it is^11^. Simple reinforcement feedback or information about correctness shows the weakest effects, whereas high-information feedback shows the strongest effects on learning outcomes^2,11^. Another influential factor on the effectiveness of feedback is the intended function of the feedback. Feedback function concerns how the feedback influences student learning and may be either cognitive by supporting learning and performance through task-related information (e.g., providing information that helps learners process task-relevant material), motivational-affective by enhancing motivation and positive emotions (e.g., encouraging effort and persistence), or self-regulatory (metacognitive) by fostering learners’ ability to plan, monitor, and evaluate their own learning (e.g., prompting learners to reflect on their performance or to evaluate their understanding)^12,13^. Apart from these content- and function-related aspects, the formal presentation mode of feedback also matters^13^: Feedback presentation can, for example, differ in timing or frequency^12^. There are only few studies examining the effect of feedback timing on feedback effectiveness, and they show no consistent advantage or disadvantage for immediate versus delayed feedback, with outcomes depending on contextual and implementation factors^15^. The frequency of feedback interventions can also be seen as a relevant moderator of its effectiveness, with meta-analytic results suggesting that frequent feedback can sometimes enhance performance, but effects vary depending on the study context and task characteristics^7^. Too frequent exposure to failure feedback may even have detrimental effects, as it can undermine learners’ feeling of control and trigger negative emotional reactions^16^. Empirical work demonstrates that feedback frequency follows an inverted-U pattern, where overly frequent feedback can overload cognitive resources and hinder performance^17^. Taken together, these findings indicate that feedback characteristics, especially the information it contains and the way it is presented, are highly relevant to understand differences in feedback effectiveness. These facets therefore provide a useful structure for studying variation in feedback strategies in technology-based learning systems.

A second line of research highlights the importance to consider not only characteristics of the feedback, but also the characteristics of the learners. Researchers emphasize the need to better understand the individual-level factors that might moderate the impact of feedback^9^. Daumiller and Meyer^18^ highlight this point, arguing that feedback should be viewed as co-constructed between the provider and the receiver, with learners’ traits playing a central role in determining whether feedback supports or undermines performance. On the one hand, learners’ cognitive prerequisites are relevant for understanding how they perceive and use feedback. The expertise-reversal effect^19^ shows that low-knowledge learners profit from high levels of guidance, whereas more knowledgeable learners benefit more from reduced assistance^20^. On the other hand, motivational-affective processes are important, as they can influence whether and how feedback is processed, and whether learners are willing to engage with it and use it effectively^21^. Pekrun’s control-value theory^16^ provides a useful framework for understanding the affective processes that connect feedback and performance. According to this theory, learners’ achievement emotions result from how much control they believe they have over a task and how much value they assign to it^16^. Feedback can directly shape both of these appraisals by shaping retrospective evaluations of past outcomes and providing cues about the probability of future success or failure. In this way, feedback affects learning outcomes through a process in which these appraisals cause certain achievement emotions, which subsequently influence performance^16^. Moreover, emotions not only follow from but also influence the processing of feedback. Whereas positive emotions such as enjoyment (activating) can foster flexible strategy use and self-regulation, negative achievement emotions such as boredom (deactivating) or anxiety (activating) may lead to superficial processing of feedback^16^. Consistent with this perspective, Fong and Schallert^21^ emphasize the role of “affective precursors”, meaning that learners’ emotional states at the moment of receiving feedback, shape their receptiveness and the degree to which feedback leads to improvement. Empirical studies support this assumption, showing that affective states can shape how learners process task information and feedback signals^22,23^. These findings suggest that both cognitive prerequisites and momentary emotional states can shape how feedback is interpreted and used. This underscores the need for feedback systems that respond to differences between and within learners rather than providing uniform feedback to all.

Technology-based learning environments are consequently placing increasing focus on adapting feedback characteristics to individual learner characteristics when trying to increase the effectiveness of feedback. Especially in such digital environments, automated feedback is expected to accurately trace learners’ activity or individual characteristics, and generate effective personalized feedback based on this information^14^. Personalizing feedback is considered a central skill of expert human teachers to help students overcome struggles and improve the learning process^4,24^. For example, Wischgoll et al.^25^ demonstrate that when teachers provide contingent, adaptive failure feedback after student errors by diagnosing misunderstandings and tailoring support before fading it, students are more likely to reconstruct their understanding and transfer learning successfully. As adaptive teachers tailor instruction to match learners’ varying needs and circumstances^26^, adaptive automated feedback must also consider many learner and situational variables, which is challenging^14^. As recent reviews emphasize, such adaptivity in technology-based learning environments remains limited, as most systems adapt feedback based on correctness or prior achievement, but only rarely take learners’ motivational or emotional states into account^5^. Research on ITS, for example, highlights that the computer-based learning environments can lead to moderate to strong improvements in learning outcomes compared to traditional instruction and, in some cases, their effectiveness is even similar to that of human tutoring^3,27,28^. However, these gains are typically achieved by adapting feedback to indicators such as learners’ current knowledge state, task performance, or response patterns. Although there is an increasing interest in creating technologies that also consider emotional aspects^29^, existing automated feedback systems that provide individualized feedback mostly rely on predefined rules or surface-level performance data and only few incorporate deeper, data-driven personalization^1^. Only about one third (34.3%) of systems adapt feedback to students’ individual characteristics, while nearly half (48%) adjust feedback solely to the task^1^. To achieve such personalization, automated feedback can be adapted in different ways, ranging from immediate, task-level feedback to broader progress-oriented guidance over time^5^. Such adaptivity can target the timing, amount, or type of the automated support, and is most effective when it encourages reflection rather than merely correcting errors^30^. Yet, the mechanisms through which adaptive feedback exerts its effects on learning are not yet fully understood, particularly with regard to how cognitive and emotional processes interact in real time to shape its effectiveness. Addressing this gap requires going beyond simply examining learning outcomes and instead, it is necessary to examine in real-time how learners process and respond to feedback, to ultimately determine whether and how adaptive feedback best supports learning. A stepwise comparison of fixed feedback delivery, frequency-adaptive feedback, and feedback that is both frequency-adaptive and personalized in content therefore offers a structured way to examine how different layers of adaptivity contribute to feedback effectiveness.

In this study, we examined the role of feedback on learners’ performance in a technology-based interactive learning environment. Following the theoretical and empirical background, and in line with Narciss^14,31^, this study treats feedback as a multidimensional construct and varies three facets: its function (cognitive or metacognitive), its content (generic or personalized information based on specific errors and prior learning steps), and its presentation (fixed or adaptive frequency). Drawing on this framework, the present study focuses on how variations in these characteristics shape learning alongside learners’ cognitive and emotional characteristics. For this purpose, we conducted an experiment in which learners received automated failure feedback (i.e., feedback after incorrect responses) while working on a cognitive learning task that involved interpreting foreign-language instructions and physically placing objects in a room. Failure feedback, delivered through a humanoid robot, was provided after incorrect placements, but its characteristics varied across three experimental conditions. In the fixed guidance condition (condition 1), failure feedback was delivered after every incorrect response (fixed frequency), resulting in a constant feedback frequency across incorrect responses (100%). In the basic-adaptive condition (condition 2), failure feedback was delivered adaptively across incorrect responses depending on learners’ recent cognitive and emotional states (adaptive frequency; ranging from 0% to 100% based on learners’ task performance and self-reported on-task enjoyment). In the personalized-adaptive condition (condition 3), the same adaptive frequency delivery strategy was applied (0–100%), but additionally the content of failure feedback was personalized to each learner’s mistakes and previous steps in the system during learning task engagement (e.g., highlighting repeated errors or referencing earlier steps in the task; personalized content). Thus, the feedback content (generic or personalized feedback) and its presentation (fixed or adaptive frequency) were systematically varied between conditions. Additionally, independent of condition, failure feedback also varied in function type, with half of all prompts being cognitive (e.g., supporting the organization and elaboration of task information), and the other half metacognitive (e.g., encouraging learners to monitor and evaluate their problem-solving strategies). Learners’ received these failure feedback prompts after incorrect placements throughout the 30-minutes interactive learning session, during which they repeatedly placed objects in the room and gradually deduced the meanings of the instructions to find each objects correct position. The session was structured into five-minute blocks, with self-reported emotional states (enjoyment and boredom) collected at the beginning of each time block. This design resulted in a three-level data structure, with placements (Level 1) nested within time blocks (Level 2), which were in turn nested within learners (Level 3). This study design allowed us to investigate how both feedback characteristics and learner characteristics influence the effectiveness of cognitive and metacognitive failure feedback on task performance, and, as not every incorrect placement triggered a feedback message, to make comparisons between trials with failure feedback and trials with no failure feedback.

Specifically, we aimed to investigate whether failure feedback improves task performance compared to receiving no failure feedback (RQ1), and how the adaptivity of frequency and personalization of content of failure feedback moderate its effectiveness (RQ2). Further, we investigated whether individual learner characteristics, such as cognitive ability and emotional states, influence how failure feedback affects task performance (RQ3). Building on the feedback intervention theory^7^, Pekrun’s control-value theory^16^, and the expertise reversal effect^19^, we hypothesized that both cognitive failure feedback (H1a) and metacognitive failure feedback (H1b) would increase the likelihood of a correct response on the next placement compared to trials with no feedback. We further hypothesized that frequency-adaptive feedback (basic-adaptive condition) would have a stronger positive effect on the task performance than fixed-frequency feedback (fixed guidance condition; H2a), and that feedback with personalized content (personalized-adaptive condition) would lead to a stronger effect on task performance than content-generic feedback (basic-adaptive condition; H2b). Finally, we expected that higher cognitive ability would reduce the impact of all forms of failure feedback on performance (H3a), while higher on-task enjoyment would increase it (H3b), and higher on-task boredom would reduce it (H3c). By investigating these hypotheses and modeling these micro-level interactions, we aimed to provide insight into the underlying cognitive and affective mechanisms through which automated failure feedback shapes learning in technology-based environments in real time. Thus, the study examines whether established theoretical mechanisms generalize to learners’ moment-to-moment responses in this learning task, and whether the technology-based implementation of feedback provides additional insights into dynamic feedback effects that may have been less apparent in prior research.

## Methods

### Sample description

Participants were recruited as part of a larger experiment on adaptive guidance in a robot-supported learning task conducted between February and June 2024 in Germany. The original study included a total of 120 participants (18–60 years, *M* = 30.25, *SD* = 10.06). Because the present analyses focus on the impact of failure feedback, the 30 participants in the no-feedback control group (simple guidance condition) were omitted, leaving a sample of *N* = 90 learners distributed evenly across the three feedback conditions (30 per condition). The retained sample ranged from 18 to 59 years (*M* = 29.53, *SD* = 10.19). A total of 61 participants (67.8%) identified as female, 27 (30%) as male, and two (2.2%) as gender diverse. Gender was assessed via a single self-report item with response options male, female, diverse, or prefer not to say. Most participants were university students (71.1%) and native German speakers (84.4%). Data on race/ethnicity, community of descent, and socioeconomic status were not collected, as these variables were not directly relevant to the research questions. Participation required fluency in German, an age of at least 18 years, and no prior knowledge of Swahili. Participants were randomly assigned to conditions and provided written informed consent prior to participation. Ethical approval for this study was granted by the University of Potsdam Ethics Committee (number 78/2023). The study was not preregistered.

### Study design

**Fig. 1:**
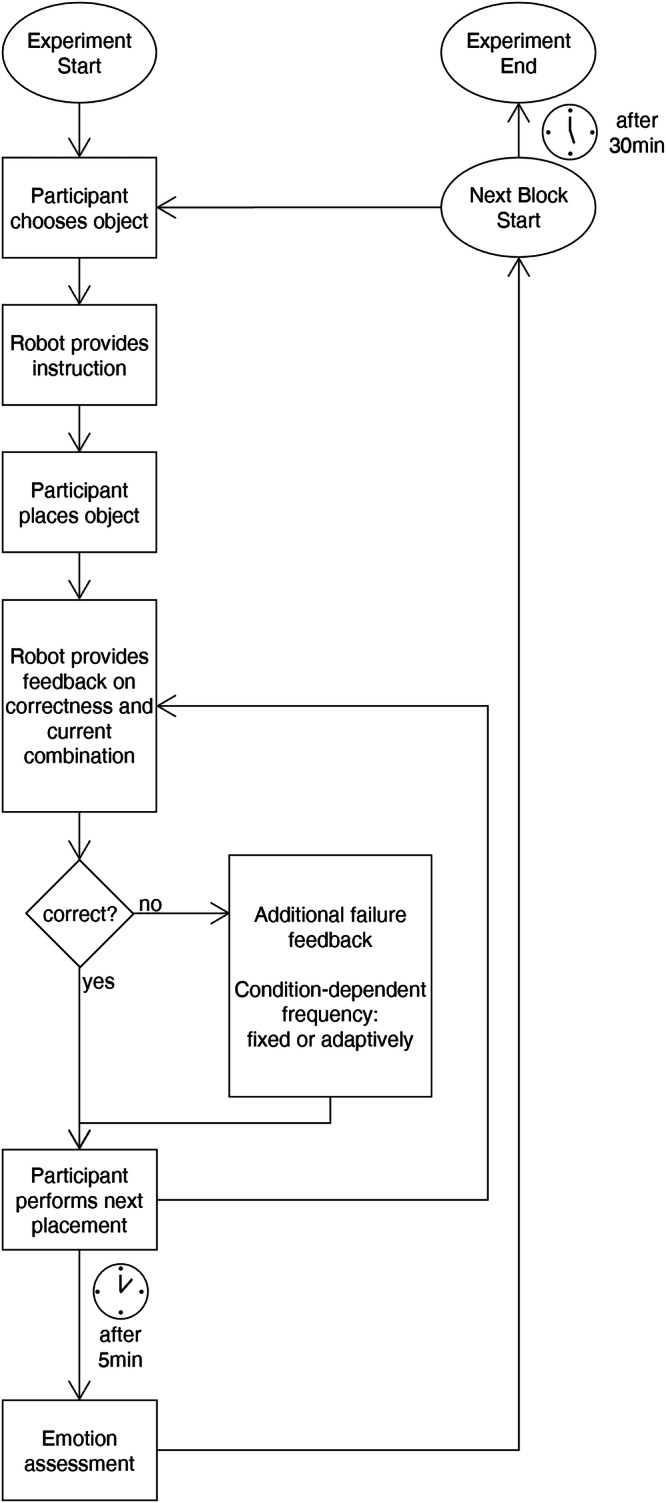
Schematic illustration of experimental flow of a single learning session*.* A learning session consisted of up to six five minutes (5-min) time blocks. Within each block, learners completed multiple trials (placements) of the vocabulary learning task. In each trial, the learner selected an object, listened to the robot’s placement instruction, and positioned the item in the room. The robot then provided feedback on correctness and the current placement combination. If the placement was incorrect, additional failure feedback could be given, with frequency determined by the experimental condition: fixed (given after every incorrect attempt) or adaptive (given selectively based on individual learner’s prior performance and most recent enjoyment rating). At the end of each 5-min block, participants rated their current emotional experience, which was used to determine the feedback strategy in the subsequent block. Trials continued until all 12 objects were correctly placed or 30 min had elapsed.

Participants interacted one-on-one with a humanoid robot in an interactive vocabulary-learning game designed to teach a selection of Swahili spatial vocabulary. The learning goal was to find the correct placements of objects in the experimental room by deducing object-location pairings from the robot’s foreign-language instructions and thereby learn the targeted vocabulary. Swahili, a language unfamiliar to the participants yet phonetically compatible with the robot’s installed German speech package, was selected to ensure both novelty and clear pronunciation. Twelve everyday objects (e.g., bottle, pen, book) lay on a central start position and 16 small white boxes marked all potential target locations in the experimental room. The number of possible locations (16) exceeded the number of objects (12) to prevent participants from solving the remaining placements by simple process of elimination toward the end of the task. For each trial the learner first freely chose an object, then listened to the robot’s instruction including the three-word goal placement in Swahili (object—preposition—location), and then physically place the object on one of the possible target locations in the room. The robot then announced in German whether the placement was correct or not and additionally repeated the Swahili phrase that described the current (incorrect) position. Depending on the experimental condition, the information about correctness was possibly followed by additional failure feedback after an incorrect placement (see Fig. 1 for a schematic illustration of the described sequence of events of a single trial). Regardless of condition, the robot always indicated whether a placement was correct or incorrect and the adaptive manipulation only determined whether an additional cognitive or metacognitive failure feedback prompt was provided additionally to the correctness feedback. After each placement, participants could freely choose the next object for the next trial. Correctly placed objects remained on their boxes, and the participant selected a new object. If a placement was incorrect, the object remained unsolved and could be attempted again in subsequent trials until it was correctly positioned. Over the course of the task, learners progressively inferred the meanings of the Swahili instructions through trial and error, leading to knowledge gained from earlier placements informing later ones. As participants attempted to place the twelve objects on their respective correct location through repeated trials, the total number of trials varied between participants depending on how quickly they solved the task. The learning phase ended when all twelve objects were correctly positioned or latest when 30 minutes had elapsed.

Before and after the interactive learning phase, participants filled out laptop-based questionnaires. Upon arrival they received written information, provided consent, and completed a pre-test that recorded sociodemographic data and included the general cognitive ability test. They then went into the interactive learning phase with the robot, which explained the game rules and guided a brief practice trial before the timed learning phase began. Every five minutes the robot paused the task to ask the learner to self-report current enjoyment, boredom, and frustration on a five-point scale via the touch screen on its chest. These emotion assessments marked the transition of experimental blocks that influenced the adaptive algorithm. After the learning session, the participant returned to the experimenter’s desk behind movable walls to complete a post-test and was debriefed before leaving the laboratory.

### Experimental manipulation of feedback characteristics

**Fig. 2:**
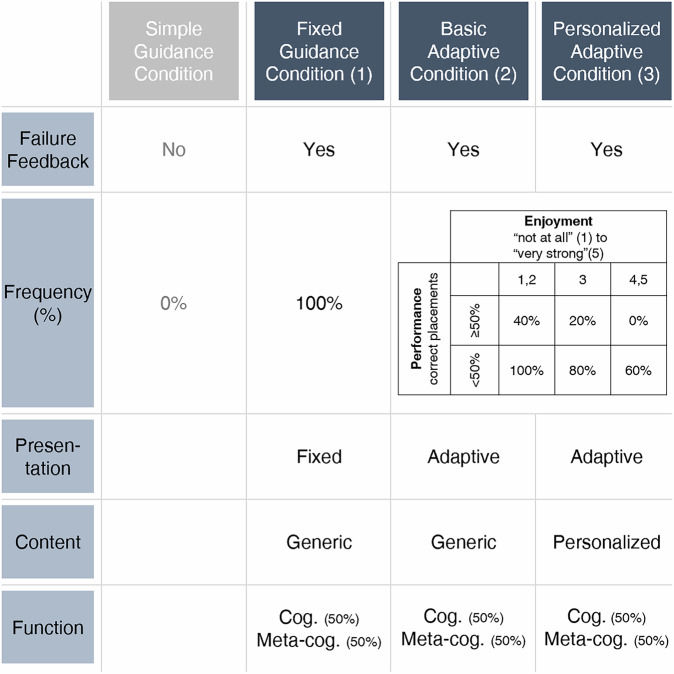
Summary of experimental conditions varying in provided failure feedback and its characteristics. The simple guidance condition, in which no failure feedback was provided after incorrect placements (0% frequency), was excluded from the present analyses. In the fixed guidance condition (condition 1), failure feedback was provided after every incorrect placement (100% frequency; fixed). In the basic-adaptive (condition 2) and personalized-adaptive condition (condition 3), feedback frequency was determined by a predefined decision matrix based on prior block performance and the learner’s most recent enjoyment rating. Higher performance reduced the probability of feedback, whereas lower enjoyment increased it. Failure feedback consisted of cognitive and metacognitive messages (50% each), selected randomly from a predefined list. In the personalized-adaptive condition, the content of these messages was personalized to the learner’s specific mistakes and learning progress.

The study employed a four-condition, between-groups design that manipulated how failure feedback was delivered following incorrect placements. A related paper using the same dataset examined general effects of adaptive guidance on learning outcomes, emotional experience, and self-regulated learning^32^, whereas the present study focuses on trial-level mechanisms of feedback effectiveness. Thus, the simple guidance condition, in which learners received only basic correctness feedback without any additional failure feedback, was excluded from the current analyses because no cognitive or metacognitive failure feedback was provided in this condition. The remaining three conditions systematically manipulated the frequency and content of failure feedback (see Fig. 2 for a visualization of experimental manipulation of failure feedback). In the fixed guidance condition (condition 1), learners received failure feedback after every incorrect placement (fixed-frequency feedback). In the basic-adaptive condition (condition 2), the frequency of failure feedback was individually adapted based on an algorithm that took into account learners’ prior block performance and self-reported on-task enjoyment (frequency-adaptive feedback). After each five-minute block, these two variables were used in a pre-defined matrix to select one out of six possible probability levels (0, 20, 40, 60, 80, or 100%). In accordance with a hint delivery strategy proposed in prior research^33^, higher prior block performance lowered that probability, while low enjoyment raised it. Prior block performance was categorized into two levels (low: < 0.50; high: ≥ 0.50), and enjoyment ratings were categorized into three levels (low: 1–2; medium: 3; high: 4–5). When prior performance was high ( ≥ 0.50), feedback probabilities ranged from 40% (low enjoyment) to 0% (high enjoyment). When prior performance was low (< 0.50), feedback probabilities ranged from 100% (low enjoyment) to 60% (high enjoyment; see Fig. 2 for the exact mapping of feedback probabilities). The resulting probability levels are descriptively provided and visualized in Supplementary Fig. 1 and Supplementary Table 1. The personalized-adaptive condition (condition 3) built on this adaptive structure by also personalizing the content of failure feedback (content-personalized feedback). In addition to adjusting how much feedback was presented (frequency), this condition adapted feedback prompts to reflect the learner’s current error and previous mistakes in the learning task (content). For instance, if a learner repeatedly misinterpreted a specific vocabulary item, the prompt highlighted this pattern to encourage targeted reflection.

Failure feedback was provided in the form of cognitively and metacognitively focused prompts. These prompts were designed either to support task-level processing (cognitive) or to foster reflection on the learner’s own learning process (metacognitive). Rather than providing explicit solutions, the failure feedback aimed to guide learners toward recognizing and addressing their own mistakes. The structure and content of the feedback were based on Zimmerman’s model of self-regulated learning^34^ and adapted from Nückles et al.^35^ to fit the demands of the specific task used in this study. Cognitive failure feedback encouraged organization and elaboration (e.g., “*How could you break the sentences into smaller parts to make it easier?*”), while metacognitive failure feedback targeted the learners’ ability to monitor and self-regulate (e.g., “*What made you choose this position? Try to reflect on your steps*”). In condition 3 (personalized-adaptive condition), the content of the failure feedback was personalized to the learner’s specific mistakes. For example, when a participant got a target location word wrong that had previously been introduced, the robot might say “*How could you break the sentences into smaller parts to make it easier? In this case, the last word could have helped you*”. Similarly, when an object was placed in an incorrect location, although based on prior information the participant should have known, the robot could respond with “What made you choose this position for *object*? Try to reflect on your steps – *you could have known it wasn’t correct*”. This personalization relied on tracking all prior placements to adapt not only the frequency but also the content of the feedback. To ensure the prompts aligned with realistic instructional practices, all feedback was carefully developed and adapted in cooperation with trained educators.

### Measures

#### Task performance

Task performance was measured at the placement level (Level 1) as a binary variable that captures whether a learner’s very next placement attempt after an incorrect placement was correct (coded as 1) or not (coded as 0). Values were computed automatically by comparing each placement with the predefined solution combination and no human rater judgments were involved. At higher levels, task performance was operationalized as aggregated means of this binary indicator across placements within time blocks (Level 2) and within learners (Level 3; overall performance).

#### Failure feedback

Failure feedback was recorded for every placement in which a learner made an error (incorrect trials). If a cognitive prompt was presented afterwards, the variable cognitive failure feedback was coded as 1 (else 0), and if a metacognitive prompt was presented, the variable metacognitive failure feedback was coded as 1 (else 0). Depending on the experimental condition, failure feedback was either always provided after errors or delivered adaptively (see Fig. 2). When failure feedback occurred, both the function type (cognitive or metacognitive) and the specific prompt were randomly selected from a predefined list, meaning that only one of the two feedback types could appear on any error trial. Across all error trials, cognitive failure feedback was delivered in 25.1% (*n* = 551), metacognitive failure feedback in 28.7% (*n* = 631), and no failure feedback in 46.2% (*n* = 1015) of cases. For statistical analyses, two binary variables were included in the model to represent the experimental manipulations of feedback frequency and content, based on the assigned condition (see section “Experimental manipulation of feedback characteristics”). Frequency was operationalized as a binary contrast between the fixed guidance condition (condition 1) and the basic-adaptive condition (condition 2). Content was operationalized as a binary contrast between the personalized-adaptive condition (condition 3) and the basic-adaptive condition (condition 2).

#### On-task emotions

Participants’ on-task emotions were measured through self-report every five minutes during the task. Using a short version of the epistemically-related emotion scale^36^ and based on control-value theory^16^, participants rated the current strength of their emotions. The self-reports were made on a five-point Likert scale ranging from “not at all” to “very strong” via the robot’s integrated touch screen. The short version uses one item per emotion, with items chosen based on high factor loadings in the full validated scale, which shows good reliability (*α* > 0.76). In this study, we focused on enjoyment and boredom because, according to control-value theory^16^, they closely relate to students’ cognitive engagement—enjoyment by promoting deep, flexible processing and boredom by indicating withdrawal. Each emotion assessment marked the start of a new experimental block, and the emotions reported immediately before each block were used as predictors in the model.

#### Cognitive ability

Learners’ general cognitive ability was measured using the short version of the Hagen Matrices Test (HMT-S)^37^, which assesses intelligent reasoning through matrix-based pattern recognition and problem-solving tasks. This brief six-item test offers an efficient measure of cognitive ability and can be completed within a few minutes. Prior research has demonstrated its reliability and validity, especially regarding its associations with academic achievement and other established intelligence measures^37^.

#### Prior block performance

Prior block performance was operationalized as the proportion of correct placements relatively to the total number of placements within the experimental block preceding the current trial. The variable ranges from 0 to 1 and values were automatically computed with no rater judgments involved.

### Statistical analysis

All hypotheses were tested in a single three-level path model (TYPE = THREELEVEL RANDOM) estimated in Mplus Version 8.10^38^ using the robust maximum-likelihood estimator (MLR) to account for non-normality. Model estimation terminated normally, and convergence criteria were met. As fit indices such as CFI and RMSEA are not available for generalized multilevel path models with binary outcomes and random slopes, model fit was evaluated using log-likelihood, AIC, and BIC values (*-2LL* = 4286.10, *AIC* = 4360.10, *BIC* = 4570.81, and sample-size-adjusted *BIC* = 4453.25). Statistical assumptions relevant for multilevel modeling were accounted for through the use of the MLR estimator, which provides standard errors robust to violations of normality and heteroscedasticity^38^. In addition, the three-level structure accounted for the hierarchical dependency of the data and thus addressed the assumption of independence of observations. Inspection of predictor correlations indicated no evidence of problematic multicollinearity ( | *r* | <0.80) for all predictors.

To assess the impact of failure feedback on task performance, the analysis focused on incorrect trials and all trials with correct placements were excluded from the dataset. The remaining trials (Level 1; *n* = 2197 incorrect placements) were nested in time blocks (Level 2; *n* = 407), which were further nested in learners (Level 3; *N* = 90). On average, participants completed 24.41 error trials (*SD* = 9.97) across 4–5 time blocks (*M* = 4.52; *SD* = 1.33). The number of trials and time blocks did not differ significantly between conditions (trials: *F*(2,87) = 1.91, *p* = 0.155; blocks: *F*(2,87) = 0.41, *p* = 0.663). The binary outcome variable was task performance, indicating whether the very next placement following an error was correct ( = 1) or incorrect ( = 0). Between levels (Level 2 and 3), this variable was aggregated accordingly to reflect average performance at the time block and learner levels. A null (intercept-only) model showed that 11.3% of the variance in task performance lay between time blocks and 0.4% between learners (ICC_BLOCK_ = 0.113; ICC_LEARNER_ = 0.004). Although the intercept ICC at the learner level is small and falls below the conventional threshold of 0.05 for meaningful between-group variance^39^, the use of a three-level multilevel model remains justified, as the central aim of the current analyses is not to explain mean-level differences in task performance, but to investigate variability in within-level random-slopes and their cross-level predictors. Importantly, intra-class correlation values are based on models with random intercepts only and do not account for variation in slopes^40^. Thus, despite minimal variance in learners’ aggregated task performance, meaningful individual differences may still exist in their responsiveness to situational predictors, such as failure feedback.

The three-level path model included a total of eight predictors across levels, included to test all three hypotheses. At the placement level (Level 1), two mutually exclusive binary variables were included as predictors of task performance. These variables coded whether a given incorrect placement was followed by cognitive failure feedback or by metacognitive failure feedback, with trials receiving no failure feedback serving as the reference. These paths estimated the direct effects of each feedback type on the likelihood of a correct placement on the subsequent trial (H1). Random slopes for both predictors were estimated and allowed to vary across time blocks (Level 2) and across learners (Level 3). At the block level (Level 2), on-task enjoyment and on-task boredom were included as block-varying predictors of the random slopes for cognitive and metacognitive feedback, to test whether on-task emotions moderated the effects of failure feedback on task performance (H3). In addition, both emotion variables were regressed on block-level aggregated task performance to assess their direct associations with average performance. Prior block performance was included as a time-varying predictor of both feedback-related slopes and of block-aggregated task performance. At the learner level (Level 3), two binary condition indicators representing the experimental manipulations of feedback characteristics were included. Frequency was represented by comparing the fixed guidance condition (condition 1) to the basic-adaptive condition (condition 2), which served as the reference group. Content was represented by comparing the personalized-adaptive condition (condition 3) to the same reference group (condition 2). Both condition indicators were included as predictors of the Level 1 feedback-related slopes, to test whether frequency and content moderated the effects of cognitive and metacognitive feedback on task performance (H2). Learners’ general cognitive ability was included as a continuous predictor of both feedback slopes to assess whether individual ability moderated the effects of failure feedback (H3). All Level 3 predictors were also regressed on learner-level aggregated task performance. All continuous predictors were grand-mean centered, while binary variables remained uncentred. Structural missing data (e.g., missing prior block performance for first blocks; *n* = 733 missing datapoints) were retained in the model and handled using Full Information Maximum Likelihood (FIML) under the MLR estimator. The outcome variable (next-trial correctness) was missing for the final incorrect trial of eight participants (*n* = 8; 0.36%) because no subsequent response existed. The model variables did not contain any missing data that was not structural.

## Results

### Descriptive statistics and correlations

Table 1 reports descriptive means and standard deviations for all study variables on their respective level, while Pearson product-moment correlations are reported separately for the three hierarchical levels in Tables 2, 3, and 4. Individual placements (Level 1; Table 2) were nested in time blocks (Level 2; Table 3), which were in turn nested in learners (Level 3; Table 4). The relation between cognitive and metacognitive failure feedback and subsequent task performance can be examined at the placement level (Table 2). Results show that metacognitive failure feedback was significantly and positively correlated with task performance (*r* = 0.11, 95% CI [0.07, 0.15], *p* < 0.001), whereas cognitive failure feedback showed no significant correlation with task performance (*r* = 0.02, 95% CI [–0.02, 0.06], *p* = 0.405). Whether feedback characteristics (conditions) were associated with learner-aggregated task performance, can be assessed on the learner level (Table 4). None of the three binary condition indicators related significantly to learner-aggregated task performance (fixed guidance condition: *r* = –0.02, 95% CI [–.22, .19], *p* = .873; basic-adaptive condition: *r* = –0.08, 95% CI [–0.28, 0.13], *p* = 0.455; and personalized-adaptive condition: *r* = 0.10, 95% CI [–0.11, 0.30], *p* = 0.364). Whether learner characteristics were associated with task performance can be examined at the block and learner levels. At the learner level (Table 4), cognitive ability was significantly and positively correlated with learner-aggregated task performance (*r* = 0.37, 95% CI [0.18, 0.54], *p* < 0.001), meaning that learners with higher cognitive ability also showed higher overall task performance. At the block level (Table 3), neither on-task enjoyment (*r* = –0.04, 95% CI [–0.13, 0.06], *p* = 0.469) nor on-task boredom (*r* = 0.03, 95% CI [–0.07, 0.13], *p* = 0.526) showed significant correlations with block-aggregated task performance.

**Table 1:** Descriptive statistics of study variables

Variable	Total sample			Condition 1			Condition 2			Condition 3			Effect Sizes		
				*Fixed Guidance Condition*			*Basic-Adaptive Condition*			*Personalized-Adaptive Condition*					
	*N*	*M*	*SD*	*n*	*M*	*SD*	*n*	*M*	*SD*	*n*	*M*	*SD*	*d*_Δ 1vs.2_	*d*_Δ 1vs.3_	*d*_Δ 2vs.3_
Level 1: variables **Placement-level**															
Task performance (0–1)	2197	0.28	0.45	793	0.27	0.45	755	0.27	0.45	649	0.31	0.46	0.00	0.09	0.09
Cognitive failure feedback (0–1)	2197	0.25	0.43	793	0.34	0.47	755	0.22	0.42	649	0.18	0.39	–0.27	–0.37	–0.10
Metacognitive failure feedback (0–1)	2197	0.29	0.45	793	0.36	0.48	755	0.26	0.44	649	0.23	0.42	–0.22	–0.29	–0.07
**Level 2: Block-level variables**															
Prior task performance (0–1)	407	0.27	0.19	141	0.26	0.19	134	0.26	0.19	132	0.28	0.18	0.00	0.11	0.11
On-task enjoyment (1–5)	407	3.39	0.88	141	3.72	0.79	134	3.26	0.95	132	3.18	0.95	–0.53	–0.62	–0.08
On-task boredom (1–5)	407	1.45	0.75	141	1.26	0.60	134	1.44	0.80	132	1.66	0.78	0.26	0.58	0.28
**Level 3: Learner-level variables**															
Cognitive ability (0–6)	90	4.21	1.27	30	4.40	1.13	30	4.07	1.23	30	4.17	1.46	–0.28	–0.18	0.07

Descriptive statistics are presented for the total sample (*N* = 90 participants) and separately for the three included conditions: Fixed guidance condition (condition 1; *n* = 30), basic-adaptive condition (condition 2; *n* = 30), and personalized-adaptive condition (condition 3; *n* = 30). Variables are presented at their respective level of the multilevel data structure. Placement- and block-level counts (*N* for total sample and *n* for conditions) may differ slightly between conditions because participants completed different numbers of trials and blocks within the fixed time window of the task. Values represent means (*M*) and standard deviations (*SD*). *d*_*Δ*_ columns report Cohen’s d effect sizes for pairwise comparisons between conditions (1 vs. 2, 1 vs. 3, and 2 vs. 3), calculated from the group means, standard deviations, and sample sizes. Effect sizes represent descriptive differences between conditions and are not inferential significance tests. The possible range for each variable is indicated in the variable labels.

**Table 2:** Bivariate correlations on the placement level (level 1)

Variable	1	2	3
1.Task performance	–		
2.Cognitive failure feedback	0.02	–	
	[–0.02, 0.06]		
3.Metacognitive failure feedback	0.11**	–0.37**	–
	[0.07, 0.15]	[–0.40, –0.33]	

Bivariate correlations (Pearson’s *r*) among placement-level study variables across all conditions. Brackets include 95% confidence intervals. * *p* < 0.05, ** *p* < 0.01.

**Table 3:** Bivariate correlations on the block level (level 2)

Variable	1	2	3	4
1.Block-aggregated task performance	–			
2.On-task enjoyment	–0.04	–		
	[–0.13, 0.06]			
3.On-task boredom	0.03	–0.43**	–	
	[–0.07, 0.13]	[–0.51, –0.35]		
4.Prior block performance	0.36**	0.16*	–0.12*	–
	[0.26, 0.45]	[0.05, 0.26]	[–0.23, –0.01]	

Bivariate correlations (Pearson’s *r*) among block-level study variables. Task performance was aggregated for each time block. Brackets include 95% confidence intervals. * *p* < 0.05, ** *p* < 0.01.

**Table 4:** Bivariate correlations on the learner level (level 3)

Variable	1	2	3	4	5
1.Learner-aggregated task performance	–				
2.Fixed guidance condition	–0.02	–			
	[–0.22, 0.19]				
3.Basic-adaptive condition	–0.08	–0.50**	–		
	[–0.28, 0.13]	[–0.64, –0.33]			
4.Personalized-adaptive condition	0.10	–0.50**	–0.50**	–	
	[–0.11, 0.30]	[–0.64, –0.33]	[–0.64, –0.33]		
5.Cognitive ability	0.37**	0.11	–0.08	–0.03	–
	[0.18, 0.54]	[–0.10, 0.31]	[–0.28, 0.13]	[–0.23, 0.18]	

Bivariate correlations (Pearson’s *r*) among learner-level variables. Task performance was aggregated for each participant. Brackets include 95% confidence intervals. * *p* < 0.05, ** *p* < 0.01.

### Impact of failure feedback on task performance (H1)

**Fig. 3:**
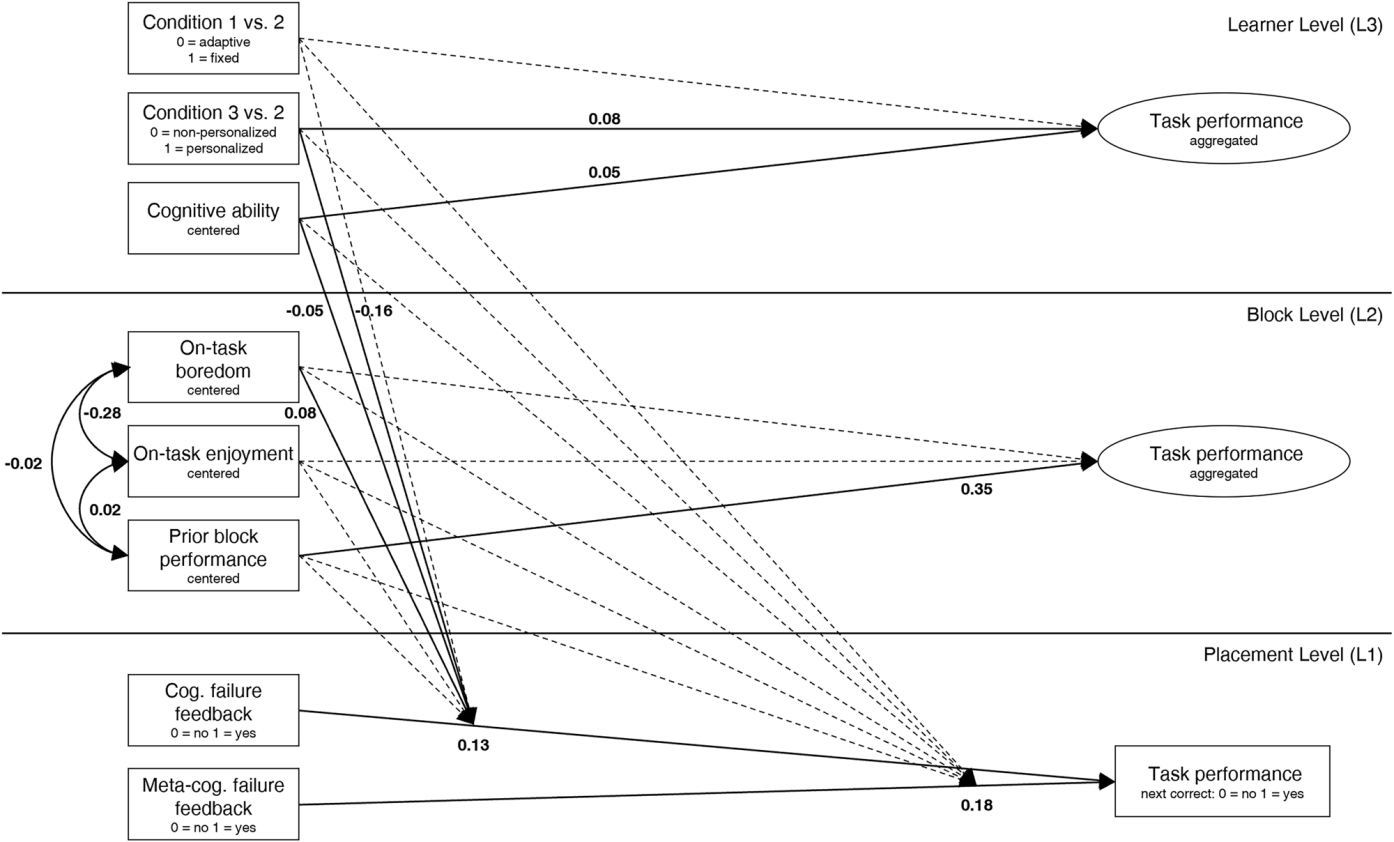
Three-level random slopes path model of feedback effects on task performance. Visualization of the tested three-level random-slopes path model including non-standardized coefficients (B). Solid, bold lines represent statistically significant coefficients (*p *< 0.05), while dashed lines indicate nonsignificant relations between constructs. Placements (Level 1; *n* = 2197) were nested in time blocks (Level 2; *n* = 407) and learners (Level 3; *n* = 90). The binary outcome, task performance, codes whether the very next placement after an error was correct (1) or incorrect (0). It was aggregated at Level 2 and Level 3 to represent block- and learner-mean performance. Feedback characteristics frequency and content are represented as binary condition indicators: Condition 1 vs. 2 compares the fixed guidance condition to the basic-adaptive condition; Condition 3 vs. 2 compares the personalized-adaptive condition to the basic-adaptive condition. All continuous predictors were grand-mean centered.

The effect of failure feedback on task performance (H1), was tested at the placement level (Level 1) of the estimated three-level random-slopes path model. All unstandardized coefficients and *p*values are reported in Table 5 and visualized in Fig. 3. Results indicate that both types of failure feedback, cognitive (*B* = 0.13, 95% CI [0.03, 0.23], *p* = 0.010) and metacognitive (*B* = 0.18, 95% CI [0.08, 0.28], *p* = 0.001), had significant and positive direct effects on task performance as compared to no feedback (see Table 5). Thus, when failure feedback followed an incorrect placement, the likelihood that the subsequent placement was correct was significantly higher than in error trials where no feedback was provided.

**Table 5:** Unstandardized results of the three-level path model

	*B*	*SE*	*95%CI*	*p*
Between-learner level (Level 3)				
Task performance (learner-aggregated)				
Condition 1 vs. 2 (fixed guidance vs. basic-adaptive condition)	–0.04	0.04	[–0.11, 0.04]	0.314
Condition 3 vs. 2 (personalized-adaptive vs. basic-adaptive condition)	0.08	0.04	[0.01, 0.15]	0.024
Cognitive ability	0.05	0.01	[0.04, 0.07]	0.000
Cognitive feedback slope (slope 1)				
Condition 1 vs. 2 (fixed guidance vs. basic-adaptive condition)	0.01	0.06	[–0.11, 0.14]	0.858
Condition 3 vs. 2 (personalized-adaptive vs. basic-adaptive condition)	–0.16	0.06	[–0.29, –0.04]	0.012
Cognitive ability	–0.05	0.02	[–0.09, –0.01]	0.008
Metacognitive feedback slope (slope 2)				
Condition 1 vs. 2 (fixed guidance vs. basic-adaptive condition)	–0.01	0.07	[–0.14, 0.12]	0.902
Condition 3 vs. 2 (personalized-adaptive vs. basic-adaptive condition)	–0.07	0.07	[–0.20, 0.07]	0.331
Cognitive ability	–0.04	0.02	[–0.08, 0.00]	0.076
Between-block level (Level 2)				
Task performance (block-aggregated)				
On-task boredom	–0.02	0.02	[–0.07, 0.03]	0.376
On-task enjoyment	–0.03	0.03	[–0.08, 0.03]	0.304
Prior block performance	0.35	0.10	[0.15, 0.54]	0.001
Cognitive feedback slope (slope 1)				
On-task boredom	0.08	0.04	[0.00, 0.16]	0.039
On-task enjoyment	0.05	0.04	[–0.02, 0.13]	0.170
Prior block performance	0.09	0.18	[–0.27, 0.45]	0.617
Metacognitive feedback slope (slope 2)				
On-task boredom	–0.01	0.04	[–0.09, 0.08]	0.898
On-task enjoyment	–0.00	0.04	[–0.08, 0.08]	0.969
Prior block performance	0.18	0.17	[-0.16, 0.51]	0.302
Within-placement level (Level 1)				
Task performance				
Cognitive failure feedback (slope 1)	0.13	0.05	[0.03, 0.23]	0.010
Metacognitive failure feedback (slope 2)	0.18	0.05	[0.08, 0.28]	0.001

Unstandardized regression coefficients (*B*) from the three-level path model are reported. *SE* = standard error; 95%*CI* = 95% confidence interval; *p* = *p*value. Data are structured hierarchically, with placements (Level 1) nested within five-minute time blocks (Level 2), which were nested within learners (Level 3). Condition comparisons reflect the experimental manipulations of feedback characteristics: Condition 1 vs. 2 compares fixed versus adaptive frequency; Condition 3 vs. 2 compares personalized versus generic content. Slopes represent cross-level moderations of the effect of cognitive (slope 1) and metacognitive (slope 2) failure feedback on task performance (Level 1).

### Moderating effects of feedback characteristics (H2)

The moderating effects of feedback characteristics (frequency and content; varied between conditions) on the relation between failure feedback and task performance (H2) were tested on the learner level (Level 3) within the same three-level model as described above (see Table 5 and Fig. 3). The cognitive and metacognitive feedback slopes represent random within-placement effects of feedback on subsequent task performance, which are allowed to vary across time blocks and learners. To assess the effect of feedback frequency on these within-level effects (H2a), condition 2 (basic-adaptive condition), in which the frequency of failure feedback was adapted based on learners’ prior block performance and on-task enjoyment ratings, was compared to condition 1 (fixed guidance condition), in which failure feedback was delivered with fixed frequency after every incorrect attempt. Results indicate that condition 2 (basic-adaptive condition) did not significantly moderate the effect of either cognitive (*B* = 0.01, 95% CI [–0.11, 0.14], *p* = 0.858) or metacognitive (*B* = –0.01, 95% CI [–0.14, 0.12], *p* = 0.902) failure feedback on task performance as compared to condition 1 (fixed guidance condition; see Table 5). Thus, the results provided no statistically reliable evidence that frequency-adaptive failure feedback influenced the likelihood that the subsequent placement was correct compared to fixed-frequency feedback. To examine the effect of feedback content (H2b), condition 3 (personalized-adaptive condition), in which feedback content was personalized to learners’ individual progress and specific mistakes in addition to adaptive frequency, was compared to condition 2 (basic-adaptive condition), in which feedback was frequency-adaptive but not content-personalized. Results showed that condition 3 (personalized-adaptive condition) had a significant and negative effect on the cognitive feedback slope (*B* = –0.16, 95% CI [–0.29, –0.04], *p* = 0.012), indicating that the effect of cognitive failure feedback on task performance was reduced for learners in condition 3 who received content-personalized feedback, compared to learners in condition 2 (basic-adaptive condition) who received content-generic feedback. However, no significant effect of condition 3 on the metacognitive feedback slope was observed (*B* = –0.07, 95% CI [–0.20, 0.07], *p* = 0.331; see Table 5).

### Moderating effects of learner characteristics (H3)

The moderating effects of learner characteristics on the relation between failure feedback and task performance (H3) were also examined within the three-level path model (see Table 5 and Fig. 3). At the block level (Level 2), on-task enjoyment and on-task boredom, both measured at the beginning of each time block, were included as block-varying predictors. On-task boredom was significantly and positively related to the cognitive feedback slope (*B* = 0.08, 95% CI [0.00, 0.16], *p* = 0.039), indicating that the performance gain from cognitive failure feedback was larger in blocks where learners self-reported higher boredom at onset. No significant effect of boredom was observed on the metacognitive feedback slope (*B* = –0.01, 95% CI [–0.09, 0.08], *p* = 0.898; see Table 5). Block-onset enjoyment did not significantly moderate the impact of either failure feedback type (cognitive feedback slope: *B* = 0.05, 95% CI [–0.02, 0.13], *p* = 0.170; metacognitive feedback slope: *B* = –0.00, 95% CI [–0.08, 0.08], *p* = 0.969). Cognitive ability, included as a continuous predictor on the learner level (Level 3), had a significant and negative effect on the cognitive feedback slope (*B* = –0.05, 95% CI [–0.09, –0.01], *p* = 0.008), indicating that learners with higher cognitive ability benefited less from cognitive failure feedback, while it did not significantly moderate the effect of metacognitive failure feedback on task performance (*B* = –0.04, 95% CI [–0.08, 0.00], *p* = 0.076; see Table 5).

### Additional findings: direct effects of moderators and control variables

In addition to the hypothesized effects, we also examined additional associations within the same three-level model (see Table 5 and Fig. 3). At the block level (Level 2), prior block performance was included as a time-varying control variable which did not significantly moderate the relation between either type of failure feedback and task performance (cognitive feedback slope: *B* = 0.09, 95% CI [–0.27, 0.45], *p* = 0.617; metacognitive feedback slope: *B* = 0.18, 95% CI [–0.16, 0.51], *p* = 0.302). Further, task performance was also included as an aggregated outcome variable at the block and learner levels (Level 2 and 3). At the block level (Level 2), prior block performance was significantly and positively associated with block-aggregated task performance (*B* = 0.35, 95% CI [0.15, 0.54], *p* = 0.001; see Table 5), indicating that better performance in the previous block predicted better performance in the current block. On-task boredom (*B* = –0.02, 95% CI [–0.07, 0.03], *p* = 0.376) and enjoyment (*B* = –0.03, 95% CI [–0.08, 0.03], *p* = 0.304) were not significantly associated with block-aggregated task performance. At the learner level (Level 3), we tested whether feedback characteristics frequency (condition 1 vs. condition 2) and content (condition 3 vs. condition 2) were directly associated with learner-aggregated task performance. Results showed that content-personalized feedback was significantly and positively associated with learner-aggregated task performance (*B* = 0.08, 95% CI [0.01, 0.15], *p* = 0.024), while frequency-adaptive feedback did not show a significant effect (*B* = –0.04, 95% CI [–0.11, 0.04], *p* = 0.314). In contrast, cognitive ability was also significantly and positively associated with learner-aggregated task performance (*B* = 0.05, 95% CI [0.04, 0.07], *p* < 0.001; see Table 5).

## Discussion

In this study, we investigated how failure feedback influences learners’ task performance in a technology-based learning environment. We examined whether failure feedback improves performance (RQ1), and how its effectiveness depends on feedback characteristics (RQ2) and learner characteristics (RQ3). Feedback characteristics (frequency and content) were manipulated across three conditions: in the fixed guidance condition (condition 1), feedback was provided after every incorrect attempt (fixed-frequency feedback); in the basic-adaptive condition (condition 2), feedback frequency was adapted to prior block performance and self-reported enjoyment (frequency-adaptive feedback); and in the personalized-adaptive condition (condition 3), both frequency and content were adapted to learners’ prior learning (content-personalized feedback). To address these questions, we tested a three-level random-slopes path model based on detailed multimodal, time-structured data, with trial-by-trial observations (Level 1) nested within time blocks (Level 2) and learners (Level 3).

The results of this study can be summarized in five key findings. First (i), both cognitive and metacognitive failure feedback significantly increased the likelihood of a correct placement in the following trial compared to error trials without failure feedback, demonstrating the general effectiveness of failure feedback in supporting immediate task performance (H1). Second (ii), frequency-adaptive feedback (condition 2) did not significantly moderate the effect of failure feedback on task performance relative to fixed-frequency feedback (condition 1), and the analyses therefore did not provide statistically reliable evidence that adjusting feedback frequency based on learners’ individual performance and on-task enjoyment influenced its immediate effectiveness (H2a). Third (iii), personalizing feedback content (condition 3) significantly reduced the positive effect of cognitive failure feedback on immediate task performance, meaning that learners in the personalized-adaptive condition benefited less from cognitive failure feedback compared to those in the basic-adaptive condition (H2b). Fourth (iv), cognitive ability significantly and negatively moderated the effect of failure feedback on performance, indicating a reduced benefit from cognitive feedback for learners with higher cognitive abilities (H3a). Fifth (v), on-task boredom significantly moderated feedback effects, showing that learners reporting higher boredom at block-onset benefited more from cognitive feedback (H3c), whereas on-task enjoyment showed no significant moderation (H3b).

### Cognitive dynamics of failure feedback effectiveness

The first finding that both cognitive and metacognitive failure feedback significantly improved learners’ immediate task performance, underscores the general value of error-contingent feedback in technology-based learning environments (H1). Although the feedback in this study solely offered cognitive and metacognitive hints that supported learners’ understanding of the task and strategy use without revealing the correct answer, it had a positive effect on learners immediate performance by increasing the likelihood of a correct response on the next attempt. This aligns with the theoretical idea from feedback intervention theory^7^ that feedback is most beneficial when it helps learners to focus their attention on task-relevant processes, as well as with empirical findings indicating that feedback in digital learning environments is most effective when it is elaborated rather than merely information about correctness^2^. Thus, this finding suggests that the applied automated failure feedback effectively guided learners back to the task and supported corrective learning after failure.

However, against our hypothesis (H2a), the effect of failure feedback on task performance was not significantly stronger for learners who received frequency-adaptive feedback compared to learners receiving this feedback on a fixed frequency level. Therefore, the results do not support the assumption derived from control-value theory^16^ that cumulative failure feedback can undermine learners’ perceived control and lead to negative achievement emotions. Based on this assumption, frequency-adaptive failure feedback was expected to support learning by reducing exposure to failure and maintaining learners’ sense of control. However, the absence of a statistically reliable effect does not necessarily indicate that frequency-adaptive failure feedback is not relevant for task performance, but rather that no effect was detected under the present conditions. In this regard, the adaptive algorithm used in this study to adjust feedback frequency may have been too coarse to capture rapid, trial-by-trial changes in learners’ performance or emotional experience that influence feedback effectiveness, given that it only updated at the block level every five minutes—representing a core limitation of this study.

Interestingly, when comparing the basic-adaptive condition and the personalized-adaptive condition, the additional personalization of the content of the frequency-adaptive failure feedback did not enhance its effect on task performance as expected (H2b), but instead significantly reduced the effectiveness of cognitive failure feedback. This suggests that tailoring feedback content to learners’ individual errors and prior learning steps did not further enhance, and may even have interfered with the immediate benefits of cognitive failure feedback. One possible explanation for this finding can be derived from cognitive load theory^41^. Personalizing the content of failure feedback resulted in prompts containing more specific information, which also increased sentence length and, consequently, the amount of information that needed to be processed. This increased processing demand may have temporarily overloaded learners’ working memory, influencing task performance on the subsequent trial. Similar findings in cognitive load research indicate that increased informational complexity of sentences in instructional material increases learners’ intrinsic cognitive load and requires greater mental effort during processing^42^. From a cognitive load perspective, this reflects the limited capacity of working memory, such that longer and more detailed feedback messages, like those used in the personalized-adaptive condition, may have required learners to process too many elements at once, thereby reducing immediate performance.

At the same time, the additional finding that content-personalized feedback was positively associated with overall task performance indicates that personalization was still beneficial at a broader level. This pattern can be interpreted through a “cost-benefit” perspective on cognitive load in digital learning environments^43^, which suggests that several design factors of digital learning technologies—including interactivity in the form of adaptive responses to learners’ actions—can induce additional, seemingly extraneous cognitive load while still enhancing learning outcomes and motivation^43^. From this perspective, the temporary processing demands caused by content-personalized failure feedback may represent a productive cognitive cost that ultimately helps the learner process information more deeply. Similar patterns have been observed in prior studies, showing that more detailed instructional feedback can initially require learners to invest greater cognitive resources but then facilitated deeper processing and integration of knowledge, resulting in improved learning performance over time^44,45^. This interpretation also aligns with the concept of desirable difficulties^46^, according to which conditions that increase processing effort can impair immediate performance while supporting learning in the long term. An alternative explanation is that personalized feedback simply increased the perceived relevance of the information by directly referring to learners’ own errors, thereby attracting greater attention and encouraging learners to relate the feedback to themselves. Research on the self-reference effect suggests that such self-related processing enhances memory as it promotes both elaboration and organization of information^47^. Thus, in contrast to more generic feedback, which may be processed more superficially, personalized feedback may have encouraged more elaborative processing, even if this temporarily interfered with immediate task performance. Taken together, these findings suggest that the observed pattern reflects a dynamic interplay between processing costs that impair immediate performance and mechanisms that support overall performance, indicating that the underlying processes are not explained by cognitive load alone and become apparent only when examined through a multi-level, situational approach of analysis.

A similar dynamic may explain why the benefits of failure feedback were significantly reduced for learners with higher cognitive ability. In accordance with our hypothesis (H3a), our fourth key finding showed that learners with higher cognitive ability benefited less from cognitive failure feedback, suggesting that the effectiveness of such automated feedback depends not only on its design but also on learners’ cognitive prerequisites. This pattern aligns with the expertise reversal effect^19^. Thus, for learners with higher cognitive ability, the elaborated cognitive feedback used in this study may have introduced unnecessary information that duplicated processes they could already perform independently. As a result, rather than improving performance, it may have increased cognitive load and interfered with their independent problem solving, showing that the same failure feedback can serve as either extraneous or intrinsic load depending on learners’ level of expertise^43^. At the same time, extending prior research, this finding indicates that the expertise reversal effect can be observed in learners’ moment-to-moment responses within a technology-based learning environment, thereby providing more fine-grained insights into how such theoretical mechanisms unfold during ongoing task performance.

### Affective dynamics of failure feedback effectiveness

But besides cognitive prerequisites, our study results further indicate that emotional learner characteristics also significantly influence the effectiveness of automated failure feedback. Specifically, learners’ situational boredom significantly moderated the effect of cognitive feedback on task performance, but contrary to our expectations (H3c), boredom increased rather than reduced its effectiveness. This finding suggests that learners who experienced higher boredom at the beginning of a block benefited more from cognitive failure feedback than those who reported lower boredom. While boredom is typically regarded as detrimental to learning within the framework of control-value theory^16^, this result suggests that failure feedback may have helped bored learners to re-engage with the task by refocusing their attention and restoring its perceived value. In contrast, learners who were less bored were likely already motivated and focused, so the failure feedback may have provided little additional benefit, resulting in smaller performance improvements. This interpretation can be based on the boredom feedback model^48^, which conceptualizes boredom as the result of inadequate attentional engagement that occurs when the desired and actual levels of attention do not match. From this perspective, the cognitive failure feedback may have functioned as a corrective signal that reduced this mismatch by refocusing attention and enhancing the perceived meaning of the task, thereby interrupting the boredom loop and facilitating re-engagement^48^. However, this moderating effect of boredom was observed only for cognitive failure feedback, as no statistically reliable moderation effect was observed for metacognitive feedback. Thus, although metacognitive processes are effortful and depend on sustained motivation and engagement^49^, and prior research on digital learning environments shows that certain emotional disruption can reduce the ability to engage in metacognitive activities^50,51^, the present data do not allow conclusions about whether these mechanisms were involved here. At the same time, the differential effects observed for cognitive and metacognitive feedback should be interpreted cautiously, as the present study did not assess whether learners perceived these feedback types as functionally distinct during the task. Although the prompts were theoretically categorized, it remains unclear to what extent learners subjectively differentiated between them during task engagement. In addition, on-task enjoyment did not significantly moderate the effect of either feedback type. Therefore, the analyses did not provide statistically reliable evidence that enjoyment influenced the effect of failure feedback on task performance. Taken together, these findings suggest that situational emotional learner characteristics can influence feedback effectiveness, although selective and depending on the type of feedback, illustrating how such affective influences become apparent at the level of learners’ moment-to-moment responses and may remain obscured in more aggregated analyses.

### Theoretical contributions

This study theoretically contributes to current research by both confirming and extending existing theories on how failure feedback functions within technology-based learning environments. By integrating perspectives from feedback intervention theory^7^, control-value theory^16^, and cognitive load theory^41^, our findings demonstrate how the situational cognitive-affective dynamics of learning shape the effectiveness of automated feedback. Using a situational approach that captures multiple levels of the learning process and possible effects in real-time, this study responds to the current growing body of research calling for more situational, temporally sensitive research on cognitive and motivational-affective processes in learning^52,53^, and contributes to opening what has been described as the “black box” of feedback interventions^6^. Findings support and extend feedback intervention theory^7^ by showing that automated failure feedback, even when not human-delivered, can effectively guide learners’ attention back to the task and improve subsequent task performance. This supports the idea that attentional redirection is a key mechanism of effective feedback and demonstrates that this can also be realized in automated systems providing pre-defined, elaborated and task-focused feedback. Building on control-value theory^16^, our results further provide empirical support for the role of emotions in feedback effectiveness. Specifically, the finding that boredom increased rather than reduced the effectiveness of cognitive failure feedback suggests that feedback can function as an immediate corrective cue that restores attention and perceived task value. This highlights that emotions such as boredom do not simply act as stable traits but dynamically interact with feedback processes, influencing performance in real time. Moreover, the study indicates that content-personalized feedback can impose cognitive demands that foster deeper processing over time. The observation that personalization of feedback content temporarily reduced feedback effectiveness but improved overall learning performance supports the idea that certain increases in cognitive load in digital learning tasks may reflect productive learning challenges rather than design flaws^43^. Taken together, these findings illustrate how established theoretical mechanisms operate at a situational level and can be more precisely understood through temporally sensitive analyses of learning processes, supporting a shift toward dynamic, context-sensitive models of feedback processes in digital learning environments.

### Limitations and future directions

Despite its theoretical contributions, this study has several limitations that should be considered when interpreting the findings. First, the study was conducted in a controlled laboratory setting with a clearly structured task and a relatively short time frame of 30 minutes. While this design allowed for precise measurement of feedback effects at the trial level, it may not fully capture the complexity of feedback processes that unfold in more authentic, long-term learning environments such as real-world classrooms. In addition, the sample consisted exclusively of adult learners, many of whom were university students. The extent to which the observed feedback dynamics generalize to younger learners or school-based educational contexts therefore remains an open question. Future studies should therefore examine how similar automated feedback mechanisms work over longer time periods, with more diverse learner populations, and across different types of learning tasks and contexts. In addition, it might be of interest to explore more learner-controlled forms of feedback delivery, for example by allowing learners to regulate the frequency of feedback themselves. Such approaches could provide insight into how externally adaptive and learner-controlled feedback mechanisms differentially affect engagement and performance. Second, although the present study employed a situational, multilevel approach to capture moment-to-moment changes in performance and emotional states, the temporal detail of the affective measures was limited. On-task emotions were assessed only every five minutes using self-report measures, which provided insight into perceived short-term fluctuations but may not have fully captured the emotional changes that occurred between individual trials. Combining such self-reports with physiological or behavioral measures could offer a more detailed insight of the emotional dynamics involved in feedback processing, for example, by capturing changes directly after failure or while receiving failure feedback. Third, the adaptivity of the feedback characteristics (frequency and content) was also implemented at the five-minute block level, meaning that adjustments were not made in real time. Although this approach allowed for integrating both cognitive and affective information into the adaptive logic, it may have been too coarse to respond to rapid fluctuations in learners’ performance or emotional states. Future studies could therefore implement more fine-grained, data-driven adaptivity that continuously adjusts feedback based on learners’ moment-to-moment behavior and affective cues. Fourth, while interpretations regarding cognitive demands are theoretically grounded in cognitive load theory^41^, they remain solely assumptions as cognitive load was not directly measured. Future research on feedback effectiveness could include multimodal measures of cognitive load to validate whether the observed effects of personalized feedback content on short-term feedback effectiveness indeed reflect temporary processing overload. In addition, systematically varying task difficulty could provide further insight into how cognitive load and learner characteristics jointly shape feedback effectiveness, for example, by examining whether the observed interaction between cognitive ability and feedback differs in more difficult versions of the task. Fifth, although cognitive and metacognitive feedback were theoretically distinguished and implemented as separate feedback functions, both types were presented in equal proportions and randomly assigned across trials. As a result, it remains unclear to what extent learners perceived and processed these feedback types as distinct signals during the task. Future research could more explicitly examine how learners differentiate between cognitive and metacognitive feedback, for example, by systematically manipulating their presentation across conditions or by assessing learners’ perceptions of different feedback functions. Finally, the feedback was delivered through a humanoid robot that served as a standardized interface for presenting the adaptive failure feedback across conditions. While this ensured experimental control, learners’ perceptions of the robot’s social presence may have influenced how feedback was received and processed. Thus, the generalizability of the findings to other contexts or feedback systems remains limited. Future research could therefore investigate how different feedback interfaces, such as virtual agents, chat-based systems, or purely text-based environments, affect the cognitive and affective real-time dynamics of automated feedback differently. In addition, future studies could systematically examine how the modality of feedback presentation (e.g., auditory, visual, or multimodal combinations of speech, text, and expressive cues) influences learners’ engagement and processing of feedback, as prior research suggests that such design features can influence learning outcomes^54^.

### Conclusion

The findings of this study provide practical implications for the design of adaptive feedback systems in technology-based learning environments. The results show that automated, elaborated failure feedback can effectively enhance learners’ immediate task performance, even when pre-defined and delivered automatically. At the same time, the finding that content-personalized feedback temporarily reduced performance but was positively associated with overall outcomes underscores the importance of balancing informativeness with cognitive load. These results suggest that feedback systems should not only adjust to learners’ performance but also consider their situative cognitive and emotional states to maintain engagement and support deeper processing over time. At the same time, results should be interpreted cautiously as the adaptive algorithm used in this study relied on relatively coarse block-level updates and a limited set of inputs. Testing these approaches in longer-term, authentic learning contexts could help translate situational insights into practical design principles for personalized feedback in digital learning environments. In doing so, this study contributes to a growing understanding of how situational cues can inform the development of adaptive technologies, while underscoring the need for further research on real-time, data-driven feedback mechanisms that can operate effectively in complex, naturalistic learning environments.

## Supplementary information

Transparent Peer Review file

Supplementary Information

## Data Availability

The datasets used and analyzed during the current study are available at https://osf.io/zungr.

## Code availability

The code used to implement the experiment is available at 10.5281/zenodo.19557127. The code used for data analyses of the present study is available at https://osf.io/zungr.
