# Supplementary material for: Real-time cognitive-affective dynamics of failure feedback in a technology-based learning task
Source: Commun Psychol. 2026 Jun 12;4:95. doi: 10.1038/s44271-026-00487-8 (PMC13263339; doi:10.1038/s44271-026-00487-8)
Supplement: Supplementary file 2 — Supplementary Information [file 44271_2026_487_MOESM2_ESM.pdf]

Real-time cognitive-affective dynamics of failure feedback in a technology-based learning task

– Supplementary Information –

Helene Ackermann[1,2,*], Anna L. Lange[2,3], Hanna Dumont[1], Verena V. Hafner[2,3], and Rebecca Lazarides[1,2]

[1]Department of Educational Sciences, Universität Potsdam, Potsdam, Germany
[2]Science of Intelligence, Research Cluster of Excellence, Berlin, Germany
[3]Department of Computer Science, Humboldt-Universität zu Berlin, Berlin, Germany
*Corresponding author: helene.ackermann@uni-potsdam.de

Supplementary Items

Supplementary Figure S1. Distribution of failure feedback probability levels across blocks and conditions.

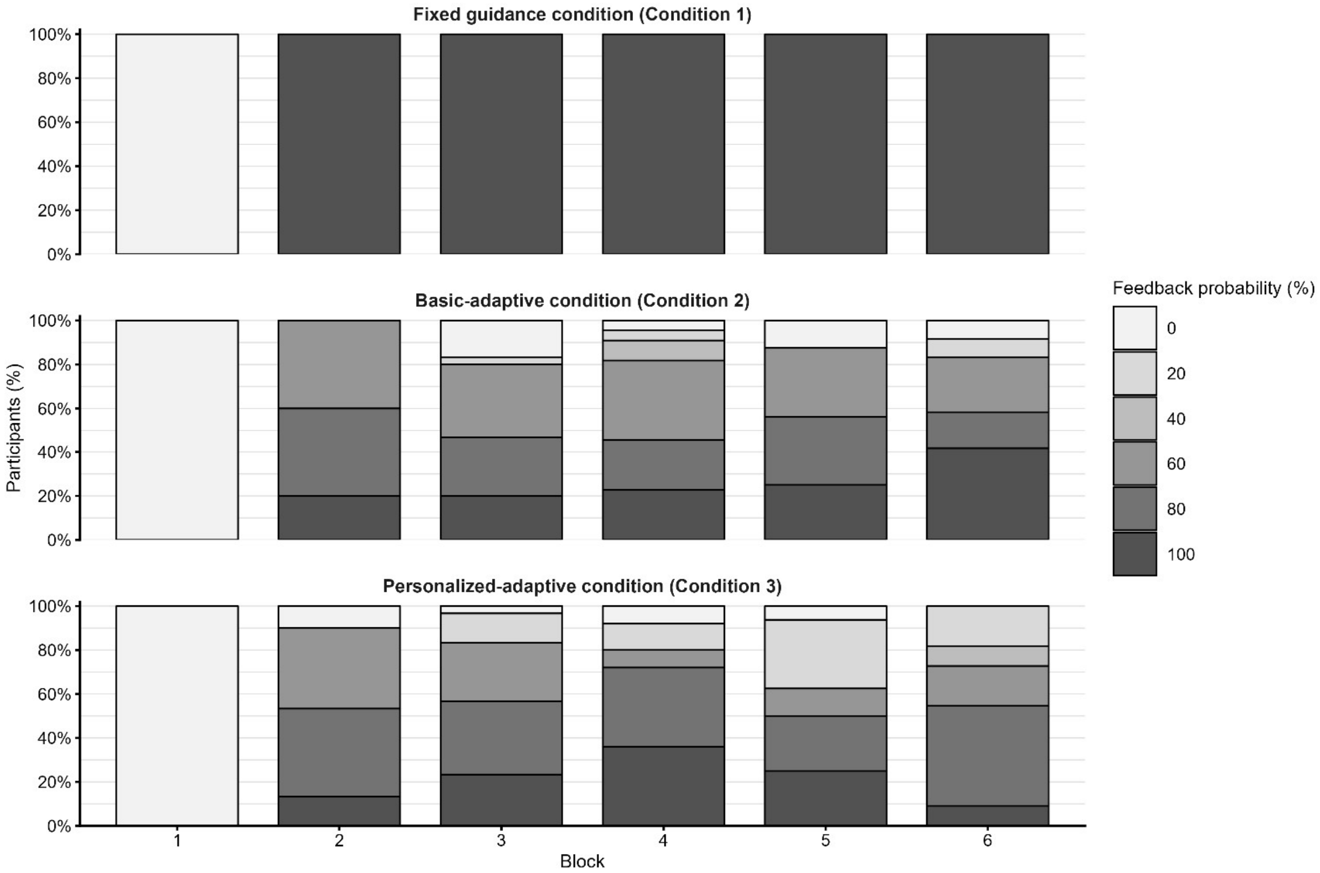

Note. Stacked bar charts show the percentage of participants assigned to each feedback probability level (0%, 20%, 40%, 60%, 80%, 100%) within each block.

Supplementary Table S1. Distribution of failure feedback probability levels across blocks and conditions.

Variable	Condition 1 *Fixed Guidance Condition*		Condition 2 *Basic-Adaptive Condition*		Condition 3 *Personalized-Adaptive Condition*	
	n	%	*n*	%	*n*	%
**Block 1 (minutes 1-5)**	**30**	–	**30**	–	**30**	–
Probability level 0%	30	100.0	30	100.0	30	100.0
Probability level 20%	0	0.0	0	0.0	0	0.0
Probability level 40%	0	0.0	0	0.0	0	0.0
Probability level 60%	0	0.0	0	0.0	0	0.0
Probability level 80%	0	0.0	0	0.0	0	0.0
Probability level 100%	0	0.0	0	0.0	0	0.0
**Block 2 (minutes 6-10)**	**30**	–	**30**	–	**30**	–
Probability level 0%	0	0.0	0	0.0	3	10.0
Probability level 20%	0	0.0	0	0.0	0	0.0
Probability level 40%	0	0.0	0	0.0	0	0.0
Probability level 60%	0	0.0	12	40.0	11	36.7
Probability level 80%	0	0.0	12	40.0	12	40.0
Probability level 100%	30	100.0	6	20.0	4	13.3
**Block 3 (minutes 11-15)**	**30**	–	**30**	–	**30**	–
Probability level 0%	0	0.0	5	16.7	1	3.3
Probability level 20%	0	0.0	1	3.3	4	13.3
Probability level 40%	0	0.0	0	0.0	0	0.0
Probability level 60%	0	0.0	10	33.3	8	26.7
Probability level 80%	0	0.0	8	26.7	10	33.3
Probability level 100%	30	100.0	6	20.0	7	23.3
**Block 4 (minutes 16-20)**	**26**	–	**22**	–	**25**	–
Probability level 0%	0	0.0	1	4.5	2	8.0
Probability level 20%	0	0.0	1	4.5	3	12.0
Probability level 40%	0	0.0	2	9.1	0	0.0
Probability level 60%	0	0.0	8	26.4	2	8.0
Probability level 80%	0	0.0	5	22.7	9	36.0
Probability level 100%	26	100.0	5	22.7	9	36.0
**Block 5 (minutes 21-25)**	**20**	–	**16**	–	**16**	–
Probability level 0%	0	0.0	2	12.5	1	6.2
Probability level 20%	0	0.0	0	0.0	5	31.2
Probability level 40%	0	0.0	0	0.0	0	0.0
Probability level 60%	0	0.0	5	31.2	2	12.5
Probability level 80%	0	0.0	5	31.2	4	25.0
Probability level 100%	20	100.0	4	25.0	4	25.0
**Block 6 (minutes 26-30)**	**12**	–	**12**	–	**11**	–
Probability level 0%	0	0.0	1	8.3	0	0.0
Probability level 20%	0	0.0	1	8.3	2	18.2
Probability level 40%	0	0.0	0	0.0	1	9.1
Probability level 60%	0	0.0	3	25.0	2	18.2
Probability level 80%	0	0.0	2	16.7	5	45.5
Probability level 100%	12	100.0	5	41.7	1	9.1

Note. Values show counts (n) and percentages (%) of participants per block and condition at each feedback probability level. Bold n values reflect participants contributing data in that block; decreases across later blocks indicate task completion. Percentages are calculated relative to the number of participants contributing data in each block.
