# Supplementary material for: Real-time cognitive-affective dynamics of failure feedback in a technology-based learning task
Source: Commun Psychol. 2026 Jun 12;4:95. doi: 10.1038/s44271-026-00487-8 (PMC13263339; doi:10.1038/s44271-026-00487-8)
Supplement: Supplementary file 1 — Transparent Peer Review file [file 44271_2026_487_MOESM1_ESM.pdf]

nature portfolio

Peer Review File

Real-time cognitive-affective dynamics of failure feedback in a technology-based learning task

Corresponding Author: Ms Helene Ackermann

This file contains all editorial decision letters in order by version, followed by all author rebuttals in order by version.

Version 0:

Decision Letter:

** Please ensure you delete the link to your author homepage in this e-mail if you wish to forward it to your coauthors **

Dear Ms Ackermann,

Thank you for your patience during the peer-review process. Your manuscript titled "Real-time cognitive-affective dynamics of failure feedback in a technology-based learning task" has now been seen by 3 reviewers, and I include their comments at the end of this message. They find your work of interest but raised some important points. We are interested in the possibility of publishing your study in Communications Psychology, but would like to consider your responses to these concerns and assess a revised manuscript before we make a final decision on publication.

We therefore invite you to revise and resubmit your manuscript, along with a point-by-point response to the reviewers. Please highlight all changes in the manuscript text file.

Editorially, we encourage you to further highlight the theoretical contribution of your work, as raised by R1. In addition, please provide sufficient methodological details and a clearer interpretation of the results, as suggested by both R2 and R3.

Please ensure you follow our statistical guidelines when reporting statistics (https://www.nature.com/commspsychol/submit/submission-guidelines#statistical-guidelines). Please note in particular our requirements for the reporting and interpretation of null-results. Non-significant findings derived from null-hypotheses significance tests should be reported in full, but may not be interpreted. Where you interpret null results, this interpretation must be based on Bayes Factors or equivalence tests.

As you revise the manuscript in response to these issues, please also implement all requests in the attached Mandatory Revision Requests document. All requirements listed in this document need to be fully met, or the work will be returned to you for further revisions without peer review. This workflow is in place to increase the likelihood that the paper will be accepted for publication. It reduces the number of rounds of revision (and review) and ensures that the reviewers vet a version of the article that is compliant with journal policies. If you have any questions regarding the required revisions, please contact the journal prior to resubmission to avoid a negative outcome.

We are committed to providing a fair and constructive peer-review process. Please don't hesitate to contact us if you wish to discuss the revision in more detail.

Please submit the following items:

- Revised manuscript
- Point-by-point response to the referees' comments
- Mandatory Revision Requests Table (attached).
- Cover letter (as a separate document)

via this link: Link Redacted .

** This url links to your confidential home page and associated information about manuscripts you may have submitted or are reviewing for us. If you wish to forward this email to co-authors, please delete the link to your homepage first **

We hope to receive your revised paper within 8 weeks; please let us know if you aren't able to submit it within this time so that we can discuss how best to proceed. If we don't hear from you, and the revision process takes significantly longer, we may close your file. In this event, we will still be happy to reconsider your paper at a later date, provided it still presents a significant contribution to the literature at that stage.

We look forward to seeing the revised manuscript and thank you for the opportunity to review your work.

Best regards,

Troby Lui, on behalf of

Yafeng Pan

Troby Lui, PhD
Associate Editor
Communications Psychology

Yafeng Pan, PhD
Editorial Board Member
Communications Psychology
orcid.org/0000-0002-5633-8313

REVIEWER EXPERTISE:
Reviewer #1: human-robot interaction, feedback, technology-based learning
Reviewer #2: human-robot interaction, feedback, technology-based learning
Reviewer #3: human-robot interaction, feedback, technology-based learning

REVIEWER REPORTS:

Reviewer #1 (Remarks to the Author):

This manuscript addresses an important and timely topic: how learners cognitively and affectively process automated failure feedback in technology-based learning environments. The use of fine-grained, trial-level data and multilevel modeling represents a valuable contribution. However, while the study is thoughtfully designed and clearly written, several conceptual, methodological, and interpretational issues currently limit the strength and generalizability of the conclusions. I therefore recommend major revision. My main concerns and suggestions are detailed below.

1. While integrating feedback intervention and control-value theories, the manuscript needs to clearly distinguish confirmation from extension. Several findings (e.g., expertise-reversal effects) align closely with existing literature; the authors should articulate what is theoretically novel in this specific technological context.
2. Cognitive and metacognitive feedback were delivered in equal proportions (50% each) and randomly selected. This randomization raises concerns about whether these distinct constructs can be functionally differentiated in the analysis. Were learners able to perceive these varying messages as distinct signals?
3. The finding that personalized feedback reduced immediate performance but improved aggregated performance is intriguing. While the authors briefly mention cognitive load, this "desirable difficulty" effect warrants a deeper discussion. Are there competing hypotheses beyond cognitive load that could explain this trade-off?
4. Regarding the null effects of frequency-adaptive feedback, the authors acknowledge the algorithm's coarseness in the Discussion. However, the Abstract and Conclusion should more explicitly frame this as a limitation of the specific algorithm used, rather than suggesting a general ineffectiveness of adaptivity based on enjoyment/performance.
5. To ensure replication, please provide the exact decision rules/matrix for the frequency-adaptive condition.
6. Please further delineate the boundaries of generalization, particularly regarding the specific age group (adults) and the nature of the learning task strictly within a controlled lab setting versus real-world classrooms.

Reviewer #2 (Remarks to the Author):

I appreciated reading this innovative paper. The study examines the effects of adaptive feedback on learning and takes

individual learning characteristics into account. The nature of the study offers many starting points for further research, and the results are very interesting and relevant both on a theoretical level for real-time insights into the processing of feedback and on a practical level for the development of intelligent tutorial systems, which are becoming increasingly important in education. I think the manuscript is very interesting for the feedback community and a wilder field of AI in education.
The introduction and discussion are very well written and coherent, covering the important and relevant theoretical background and painting a coherent picture. There are still a few points that could be improved in the results section.
Below, I highlight specific sentences and sections with remarks and comments that need to be revised.
- The last sentence in the abstract is rather unspecific. You might formulate a more meaningful sentence.
- I was wondering if you have an ethical votum and a preregistration for this study.
- In the section starting at line 147, I would appreciate it if a little more information were provided about the characteristics of the feedback in order to gain a better understanding, especially since the methods are only described at the very end of the paper and it is otherwise difficult to understand the results. (see also the next three points)
o The definition and the difference of fixed and adaptive frequency could be explained in more detail. Reading it for the first time, I was not sure what exactly is meant by this. Fixed feedback could be also associated with content as giving every time failure feedback regardless of the correctness of the answer. So, the word “fixed” can easily be associated with content. Maybe it would help to make this more explicit.
o A few more information on how the feedback looks like would be helpful, especially what adaptive frequency means. What is the range – how often was adaptive feedback given?
o It would also be helpful to add here if the tasks of the vocabulary learning game build on each other or are independent from each other.
- In the whole result section, the references for tables and figures are missing. Please insert them in every section at the respective places.
- The lines 204-209 do not contain the results for H1. I would recommend placing this general section above line 203 to make the structure clearer.
- Please check the labels of the conditions in Table 3 and the result section, especially for H2 (ll. 214-233). The labels do not correspond exactly, which is a bit confusing.
- I am not sure if the interpretation on the results for H2 in the discussion can really been explained by the cognitive load theory. There would need to be a big difference in the amount or the characteristics of feedback between the adaptive and the personalized-adaptive condition. Are there for example more words used for the personalized-adaptive feedback compared to the adaptive feedback?
- As you mentioned in the sample description, that the no-feedback condition was not used for this paper, I was wondering if there are any other related papers, which use the no-feedback condition or if this study was part of a bigger project. If this is the case, please mention this in the method section and indicate any publications or pre-registrations relating to the paper.
- I am not sure what exactly happened in the adaptive frequency condition when no feedback is given. Was it indicated that the answer was correct or incorrect or did the participants directly answer the next question?
- Please indicate how many datapoints were replaced by FIML (l. 581)?
- In Table 1, if find the different values without labeling a bit confusing. Can you indicate which numbers represent cases and which numbers represent participants.
- I was also wondering if mean and SD of task performance for Condition 1 and 2 are exactly the same in Table 1.
- In Figure 1 the dashed lines are not clearly visible in a printed version. It might be useful to use bold dashed lines.

Reviewer #3 (Remarks to the Author):

Summary:
Testing adults, the authors were interested in determining how the type of feedback influenced learning for a technology based task. The authors state that while automated feedback for technology-based learning environments is growing in popularity, the true effectiveness of these systems is not known due to variations in type of feedback used in previous studies and the lack of information available to assess how learners perceive feedback. To address these gaps in the literature, the authors analyzed a dataset from a previous experiment with a vocabulary learning game in which the function, content, and presentation of feedback from a robot were controlled. The first condition had “fixed” guidance for every incorrect response, the second condition was the “basic-adaptive” feedback condition which measured task performance and enjoyment, the third condition was the “personalized-adaptive” condition where both frequency and content were adaptive. Regardless of the conditions, half of the feedback prompts were cognitive and the other half were metacognitive. Fluent German speakers were recruited as participants and used the touch screen to move objects to a location. Objects and the location were presented to the participants in Swahili, feedback for incorrect trials (placements) was provided in German. A five-point Likert scale was used to capture self-reported emotion assessments (boredom and enjoyment) every 5 minutes. Their results showed that metacognitive feedback was significantly related with task performance (correcting a previously incorrect placement of an object), while cognitive feedback showed no correlation. They also found that learners with higher cognitive ability had better performance. Emotional assessments were not significantly correlated with block-aggregated task performance. A model testing the impact of feedback type on performance revealed that both cognitive and metacognitive feedback types had significant positive effects on performance. A separate model revealed that feedback frequency did not predict performance, so there was no difference in fixed vs basic-adaptive conditions. Another model revealed that even though both cognitive and metacognitive feedback types impacted performance, participants in the personalized-adaptive condition surprisingly benefitted less than basic-adaptive feedback for cognitive feedback: people with generic feedback performed better for cognitive feedback. Learners improved more with cognitive feedback in blocks with higher self-reported boredom while enjoyment was not significant. Learners with higher cognitive ability benefited less

from cognitive feedback. The study results are interesting, and will be useful for people interested in measuring the effects of feedback, especially future studies involving imaging tools and data driven methods.

Overall Review: This manuscript is very applicable and presents many interesting questions about the quality of feedback in relation to performance. While the study is good, it would be greatly improved with some revisions, particularly by (1) adding clarification details for the methods that allow readers to replicate the study and (2) making the results as interpretable as possible for readers.

Comments by Section:

Introduction
1. Line 48: please define meta-task processes
2. Line 76: please provide an example of "metacognitive" functions of feedback. You do this in the methods, but it would be helpful for the readers to have some sense of a difference between cognitive and metacognitive feedback functions when you describe the procedure in Line 168. Also, please be consistent in how metacognitive is used, either "meta-cognitive" or "metacognitive".

Results
3. Line 693, Table 1: For the effect sizes, can you change the font style to bold for the row values based on whether or not the differences between factors (1 vs 2, 1 vs 3, and 2 vs 3) were statistically significant?
4. Line 214 - 258: For the results mentioned in the "Moderating effects of feedback characteristics (H2)", "moderating effects of learner characteristics (H3)" and "additional findings: direct effects of moderators and control variables", are any of the results presented in the three tables or three figures? If so, can you include a reference to the appropriate table for each result in the text? For these sections, can you also consider including the mean differences and p values for the comparisons in the text directly?

Discussion
5. Line 294 - 296: Did the authors consider a condition for a future study in which participants had control over feedback frequency? This would be less automatic, but users would have more control.
6. Line 331 - 335: The finding that people with higher cognitive ability benefited less from feedback is interesting. Did the authors consider how cognitive ability would interact with performance for a more difficult but similar vocabulary task? A future study could test how feedback, cognitive ability, and task difficulty interact with performance for advanced learners.

Methods
7. Line 441: Is it possible to show a visual representation of an example trial from the experiment as a figure in the manuscript?

8. Since the results are based on data from a previous experiment, please include the month(s) and year(s) in which the original data from the experiment was collected. If possible, include a reference to the published data so readers can check if they are interested in reading about the previous study.

9. Line 453: On a related note to the previous comment, I was left wondering how the technology-based vocabulary experiment was controlled? Did participants see both a computer monitor and the robot or was the robot an all-in-one machine with a touch screen display? What is the physical size, resolution and sampling rate (Hz) of the display screen used for testing? How far did participants sit away from the robot and or screen? Please list the name of the software that controlled the procedure and/or controlled the vocabulary game. Please clarify how participants made their response for the learning phase. Based on what was described in the manuscript, I am assuming participants used their fingers for a touch and drag response to move the object, but was wondering if participants had to use the mouse or keyboard for any responses. Readers may want to try to use the software/game in future studies and this detail will allow for better replication. If these details are already stated in a different article that had the original data, then you can point the reader to that article instead.

10. Line 441: Is the reason for there being more locations (16) than objects (12) so that participants cannot rely on the process of elimination for the last few incorrect targets? If so, this detail should be included in the methods.

11. Line 449: "The learning phase ended when all twelve objects were correctly positioned or when 30 minutes had elapsed." This makes it sound like each participant completed only 12 trials. At Line 469, the manuscript says "after each five-minute block", which sounds like there were 12 trials per block that ended after all responses were corrected or after 5 minutes had elapsed. Did each participant complete 6 blocks? Either way, please update the sentence at line 449 so readers know how many trials and blocks were completed by each person in the entire experiment.

12. Line 469-470: The "pre-defined matrix to select one out of six possible probability levels" is a good way to gauge performance and emotional assessment across time. Readers might be interested in knowing the distribution of each probability level for the adaptive conditions. Can you report the percentage of blocks in which participants had a probability level of 0, percentage of blocks when participants had a probability of 20, 40, 60, 80, and 100%? This would be useful for highlighting task difficulty and learning between blocks. You could also look at block order: based on your hypotheses, you might expect to see increased probabilities (80% or higher) for Block 1 due to low performance and lower probabilities (20% or lower) for Block 6 due to better performance and learning for the task. This would be similar to the trial error statistics mentioned in lines 509 - 510 except applied to the probability levels for the adaptive conditions.

Other Comments
13. The study design section says that participants “listened to the robot’s instruction” and that the “robot announced” the feedback in German. Given that the authors were interested in testing how different types of feedback influenced performance, did the authors consider how audio feedback would differ from visual feedback? Such as presenting the feedback as text on the touch screen instead of audio, or both. Or testing different types of the robot’s facial expressions. These ideas could be included along with the suggestions in line 410 - 412.

* TRANSPARENT PEER REVIEW: Communications Psychology uses a transparent peer review system. This means that we publish the editorial decision letters including Reviewers' comments to the authors and the author rebuttal letters online as a supplementary peer review file. However, on author request, confidential information and data can be removed from the published reviewer reports and rebuttal letters prior to publication. If your manuscript has been previously reviewed at another journal, those Reviewers' comments would not form part of the published peer review file.

** Visit Nature Research's author and referees' website at www.nature.com/authors for information about policies, services and author benefits**

Communications Psychology is committed to improving transparency in authorship. As part of our efforts in this direction, we are now requesting that all authors identified as ‘corresponding author’ create and link their Open Researcher and Contributor Identifier (ORCID) with their account on the Manuscript Tracking System prior to acceptance. ORCID helps the scientific community achieve unambiguous attribution of all scholarly contributions. You can create and link your ORCID from the home page of the Manuscript Tracking System by clicking on ‘Modify my Springer Nature account’ and following the instructions in the link below. Please also inform all co-authors that they can add their ORCIDs to their accounts and that they must do so prior to acceptance.
https://www.springernature.com/gp/researchers/orcid/orcid-for-nature-research

For more information please visit http://www.springernature.com/orcid

If you experience problems in linking your ORCID, please contact the Platform Support Helpdesk.

Version 1:

Decision Letter:

** Please ensure you delete the link to your author homepage in this e-mail if you wish to forward it to your coauthors **

Dear Ms Ackermann,

Your manuscript titled "Real-time cognitive-affective dynamics of failure feedback in a technology-based learning task" has now been seen by our reviewers, whose comments appear below. In light of their advice I am delighted to say that we are happy, in principle, to publish a suitably revised version in Communications Psychology.

We therefore invite you to revise your paper one last time to address the remaining concerns of our reviewers and a list of editorial requests. At the same time we ask that you edit your manuscript to comply with our format requirements and to maximise the accessibility and therefore the impact of your work.

EDITORIAL REQUESTS:

Please review our specific editorial comments and requests regarding your manuscript in the attached "Editorial Requests Table". Please outline your response to each request in the right hand column. Please upload the completed table with your manuscript files as a Related Manuscript file.

If you have any questions or concerns about any of our requests, please do not hesitate to contact me.

SUBMISSION INFORMATION:

In order to accept your paper, we require the files listed here https://www.nature.com/documents/commsj-file-checklist.pdf .

OPEN ACCESS:

Communications Psychology is a fully open access journal. Articles are made freely accessible on publication. For further information about article processing charges, open access funding, and advice and support from Nature Research, please visit https://www.nature.com/commspsychol/open-access

At acceptance, you will be provided with instructions for completing the open access licence agreement on behalf of all authors. This grants us the necessary permissions to publish your paper. Additionally, you will be asked to declare that all required third party permissions have been obtained, and to provide billing information in order to pay the article-processing charge (APC).

* TRANSPARENT PEER REVIEW: Communications Psychology uses a transparent peer review system. On author request, confidential information and data can be removed from the published reviewer reports and rebuttal letters prior to publication. If you are concerned about the release of confidential data, please let us know specifically what information you would like to have removed. Please note that we cannot incorporate redactions for any other reasons.

* CODE AVAILABILITY: All Communications Psychology manuscripts must include a section titled "Code Availability" at the end of the methods section. We require that the custom analysis code supporting your conclusions is made available in a publicly accessible repository at this stage; please choose a repository that generates a digital object identifier (DOI) for the code; the link to the repository and the DOI must be included in the Code Availability statement. Publication as Supplementary Information will not suffice.

* DATA AVAILABILITY:
All Communications Psychology manuscripts must include a section titled "Data Availability" at the end of the Methods section. More information on this policy, is available in the Editorial Requests Table and at http://www.nature.com/authors/policies/data/data-availability-statements-data-citations.pdf.

Please use the following link to submit the above items:
Link Redacted
** This url links to your confidential home page and associated information about manuscripts you may have submitted or be reviewing for us. If you wish to forward this email to co-authors, please delete the link to your homepage first **

We hope to hear from you within two weeks; please let us know if you need more time.

Best regards,

Troby Lui

Troby Lui, PhD
Associate Editor
Communications Psychology

Yafeng Pan, PhD
Editorial Board Member
Communications Psychology
orcid.org/0000-0002-5633-8313

REVIEWERS' COMMENTS:

Reviewer #1 (Remarks to the Author):

I thank the authors for their thoughtful and thorough response to my previous comments. The revised manuscript shows substantial improvement in clarity, theoretical framing, and methodological transparency. In particular, the clearer distinction between findings that confirm existing theories and those that extend them effectively addresses my earlier concern. Before final acceptance, I have one remaining point that I believe would further strengthen the manuscript.

The authors now acknowledge that no manipulation check was conducted to verify whether learners perceived cognitive and metacognitive feedback as functionally distinct. This is an honest and appropriate addition. I would encourage the authors to go one small step further by noting how this limitation constrains the interpretation of the differential effects observed, for instance, the finding that boredom moderated only the effect of cognitive feedback. Readers would benefit from seeing this caveat linked explicitly to the corresponding findings in the Discussion rather than appearing only in the general limitations paragraph.

Reviewer #2 (Remarks to the Author):

Dear Authors,

thank you for your thorough response to my comments and questions. I am overall satisfied with you editing. I have just one more comment.

Regarding the changes you made in the discussion for the interpretation with cognitive load theory.
I appreciate the revised interpretation. However, I feel that the mention of CTML here is redundant and inappropriate. CTML is based on cognitive load theory, and the argument put forward is exactly the same. Furthermore, CTML does not align with the feedback characteristics, as your study involves text- / speech-based feedback only and does not use additional materials such as pictures. Please revise this section again.

Reviewer #3 (Remarks to the Author):

I have no further comments. The manuscript should be accepted.

** Visit Nature Research's author and referees' website at www.nature.com/authors for information about policies, services and author benefits**

Open Access This Peer Review File is licensed under a Creative Commons Attribution 4.0 International License, which permits use, sharing, adaptation, distribution and reproduction in any medium or format, as long as you give appropriate credit to the original author(s) and the source, provide a link to the Creative Commons license, and indicate if changes were made.
In cases where reviewers are anonymous, credit should be given to 'Anonymous Referee' and the source.
The images or other third party material in this Peer Review File are included in the article's Creative Commons license, unless indicated otherwise in a credit line to the material. If material is not included in the article's Creative Commons license and your intended use is not permitted by statutory regulation or exceeds the permitted use, you will need to obtain permission directly from the copyright holder.
To view a copy of this license, visit https://creativecommons.org/licenses/by/4.0/

Reviewer #1 (Remarks to the Author):

This manuscript addresses an important and timely topic: how learners cognitively and affectively process automated failure feedback in technology-based learning environments. The use of fine-grained, trial-level data and multilevel modeling represents a valuable contribution. However, while the study is thoughtfully designed and clearly written, several conceptual, methodological, and interpretational issues currently limit the strength and generalizability of the conclusions. I therefore recommend major revision. My main concerns and suggestions are detailed below.

We appreciate this positive acknowledgement of the manuscript's topic and contribution and highly value the helpful feedback of the reviewer.

1. While integrating feedback intervention and control-value theories, the manuscript needs to clearly distinguish confirmation from extension. Several findings (e.g., expertise-reversal effects) align closely with existing literature; the authors should articulate what is theoretically novel in this specific technological context.

Thank you for highlighting this. We revised the manuscript to more clearly distinguish between findings that confirm existing theories and those that extend them in the context of technology-based, automated feedback. In the Introduction, we now explicitly clarify that the study examines whether established theoretical mechanisms generalize to learners' moment-to-moment responses in a technology-based learning environment (lines 201-203). In the Discussion, we more clearly differentiate between confirming and extending contributions. For example, we note that the expertise reversal effect aligns with prior research while extending it by showing how it unfolds at a fine-grained, trial-by-trial level in this setting (lines 563-566). In addition, the theoretical contribution section has been revised to explicitly state that the study both confirms and extends existing frameworks (e.g., lines 601-602, 608-609, and 621-624). See, for example, the following changes:

> Introduction, lines 201-203:
> *"Thus, the study examines whether established theoretical mechanisms generalize to learners' moment-to-moment responses in this learning task, and whether the technology-based implementation of feedback provides additional insights into dynamic feedback effects that may have been less apparent in prior research."*
>
> Discussion, lines 563-566:
> *"At the same time, extending prior research, this finding indicates that the expertise reversal effect can be observed in learners' moment-to-moment responses within a technology-based learning environment, thereby providing more fine-grained insights into how such theoretical mechanisms unfold during ongoing task performance."*
>
> Discussion, lines 601-602:
> *"This study theoretically contributes to current research by both confirming and extending existing theories on how failure feedback functions within technology-based learning environments"*
>
> Discussion, lines 608-609:
> *"Findings support and extend feedback intervention theory"*

Discussion, lines 621-624:

"Taken together, these findings illustrate how established theoretical mechanisms operate at a situational level and can be more precisely understood through temporally sensitive analyses of learning processes, supporting a shift toward dynamic, context-sensitive models of feedback processes in digital learning environments."

2. Cognitive and metacognitive feedback were delivered in equal proportions (50% each) and randomly selected. This randomization raises concerns about whether these distinct constructs can be functionally differentiated in the analysis. Were learners able to perceive these varying messages as distinct signals?

Thank you for raising this important point. Cognitive and metacognitive feedback were distinguished at the level of content and implemented based on theoretical definitions. To ensure comparable exposure across participants and conditions, both feedback types were delivered in equal proportions and randomly assigned across trials. Unfortunately, we did not include a manipulation check to assess whether learners perceived these feedback types as distinct signals. We have therefore added this as a limitation in the Discussion (lines 656-661), noting that the present study cannot directly assess learners' subjective differentiation between feedback types and suggesting that future research should examine how learners perceive and interpret different feedback functions.

Discussion, lines 656-661:

"Fifth, although cognitive and metacognitive feedback were theoretically distinguished and implemented as separate feedback functions, both types were presented in equal proportions and randomly assigned across trials. As a result, it remains unclear to what extent learners perceived and processed these feedback types as distinct signals during the task. Future research could more explicitly examine how learners differentiate between cognitive and metacognitive feedback, for example by systematically manipulating their presentation across conditions or by assessing learners' perceptions of different feedback functions."

3. The finding that personalized feedback reduced immediate performance but improved aggregated performance is intriguing. While the authors briefly mention cognitive load, this "desirable difficulty" effect warrants a deeper discussion. Are there competing hypotheses beyond cognitive load that could explain this trade-off?

We have extended the discussion of this finding to include complementary explanations beyond cognitive load. Specifically, we now incorporate the concept of desirable difficulties and an additional explanation based on self-referential processing to provide a more comprehensive interpretation of the observed trade-off between immediate and aggregated performance. These revisions can be found in the Discussion (lines 533-554), where we clarify that the observed pattern may reflect a combination of increased processing demands and more elaborative, self-relevant processing, rather than being explained by cognitive load alone.

Discussion, lines 533-554:

"At the same time, the additional finding that content-personalized feedback was positively associated with overall task performance indicates that personalization was still beneficial at a broader level. This pattern can be interpreted through a "cost-benefit" perspective on cognitive load in digital learning environments[44], which suggests that several design factors of digital learning technologies – including interactivity in the form of adaptive responses to learners' actions – can induce additional, seemingly extraneous cognitive load while still

enhancing learning outcomes and motivation[44]. From this perspective, the temporary processing demands caused by content-personalized failure feedback may represent a productive cognitive cost that ultimately helps the learner process information more deeply. Similar patterns have been observed in prior studies, showing that more detailed instructional feedback can initially require learners to invest greater cognitive resources but then facilitated deeper processing and integration of knowledge, resulting in improved learning performance over time[45,46]. This interpretation also aligns with the concept of desirable difficulties[47], according to which conditions that increase processing effort can impair immediate performance while supporting learning in the long term. An alternative explanation is that personalized feedback simply increased the perceived relevance of the information by directly referring to learners' own errors, thereby attracting greater attention and encouraging learners to relate the feedback to themselves. Research on the self-reference effect suggests that such self-related processing enhances memory as it promotes both elaboration and organization of information[48]. Thus, in contrast to more generic feedback, which may be processed more superficially, personalized feedback may have encouraged more elaborative processing, even if this temporarily interfered with immediate task performance. Taken together, these findings suggest that the observed pattern reflects a dynamic interplay between processing costs that impair immediate performance and mechanisms that support overall performance, indicating that the underlying processes are not explained by cognitive load alone and become apparent only when examined through a multi-level, situational approach of analysis."

4. Regarding the null effects of frequency-adaptive feedback, the authors acknowledge the algorithm's coarseness in the Discussion. However, the Abstract and Conclusion should more explicitly frame this as a limitation of the specific algorithm used, rather than suggesting a general ineffectiveness of adaptivity based on enjoyment/performance.

Thank you for this important comment. We agree that the null effects of frequency-adaptive feedback should not be interpreted as evidence for a general ineffectiveness of adaptivity based on performance and enjoyment. We have therefore revised the Abstract, Results, Discussion, and Conclusion to more clearly constrain the interpretation of this finding. Specifically, we now consistently emphasize that the analyses did not provide statistically reliable evidence for an effect (Abstract, lines 20-21; Results, lines 428-430; Discussion, lines 482-485, 504-517). In addition, we explicitly frame the null finding as specific to the implemented adaptive algorithm. To make this clearer, we also added a statement in the Conclusion noting that the algorithm relied on relatively coarse, block-level updates and a limited set of input variables, which may have constrained its effectiveness (lines 679-680).

Abstract, lines 20-21:
"Relative to fixed guidance (condition 1), the implemented form of frequency-adaptive feedback (condition 2) did not statistically reliably moderate these effects."

Results, lines 428-430:
"Thus, the results provided no statistically reliable evidence that frequency-adaptive failure feedback influenced the likelihood that the subsequent placement was correct compared to fixed-frequency feedback."

Discussion, lines 482-485:

“Second (ii), frequency-adaptive feedback (condition 2) did not significantly moderate the effect of failure feedback on task performance relative to fixed-frequency feedback (condition 1), suggesting that the analyses did not provide statistically reliable evidence that adjusting feedback frequency based on learners’ individual performance and on-task enjoyment influenced its immediate effectiveness (H2a).”

Discussion, lines 504-517:
“However, against our hypothesis (H2a), the effect of failure feedback on task performance was not significantly stronger for learners who received frequency-adaptive feedback compared to learners receiving this feedback on a fixed frequency level. Thus, the analyses did not provide statistically reliable evidence for differences in the effect of automated failure feedback depending on whether it was delivered after every single incorrect placement or adapted in frequency to the individual learner’s recent performance and on-task enjoyment. Accordingly, this result does not support the assumption derived from control-value theory[16] that cumulative failure feedback can undermine learners’ perceived control and lead to negative achievement emotions. Based on this assumption, frequency-adaptive failure feedback was expected to support learning by reducing exposure to failure and maintaining learners’ sense of control. At the same time, the absence of a statistically reliable effect does not necessarily indicate that frequency-adaptive failure feedback is not relevant for task performance, but rather that no effect was detected under the present conditions. In this regard, the adaptive algorithm used in this study to adjust feedback frequency may have been too coarse to capture rapid, trial-by-trial changes in learners’ performance or emotional experience that influence feedback effectiveness, given that it only updated at the block level every five minutes – representing a core limitation of this study.”

Conclusion, lines 679-680:
“At the same time, results should be interpreted cautiously as the adaptive algorithm used in this study relied on relatively coarse block-level updates and a limited set of inputs.”

5. To ensure replication, please provide the exact decision rules/matrix for the frequency-adaptive condition.

To improve transparency and ensure replicability, we have revised the Methods section to explicitly describe the decision rules underlying the frequency-adaptive feedback. Specifically, we now describe in detail how prior performance and enjoyment were categorized and how these combinations determined the feedback probabilities (lines 268-273). The full mapping between these variables and the resulting probability levels is now described in the text and remains illustrated in Figure 3.

Methods, lines 268-273:
“Prior block performance was categorized into two levels (low: < .50; high: ≥ .50), and enjoyment ratings were categorized into three levels (low: 1-2; medium: 3; high: 4-5). When prior performance was high (≥ .50), feedback probabilities ranged from 40% (low enjoyment) to 0% (high enjoyment). When prior performance was low (< .50), feedback probabilities ranged from 100% (low enjoyment) to 60% (high enjoyment; see Figure 3 for the exact mapping of feedback probabilities).”

6. Please further delineate the boundaries of generalization, particularly regarding the specific age group (adults) and the nature of the learning task strictly within a controlled lab setting versus real-world classrooms.

Thank you for this helpful comment. We have revised the limitations section to more clearly delineate the boundaries of generalization with respect to both the sample and the learning context. Specifically, we now explicitly state that the sample consisted of young adult learners (predominantly university students) and clarify that the findings may not directly generalize to other age groups or school-based educational settings. We also more clearly contrast the controlled laboratory task with more complex, long-term learning processes in real-world classrooms (lines 627-634).

> Discussion, lines 627-634:
> *"First, the study was conducted in a controlled laboratory setting with a clearly structured task and a relatively short time frame of 30 minutes. While this design allowed for precise measurement of feedback effects at the trial level, it may not fully capture the complexity of feedback processes that unfold in more authentic, long-term learning environments such as real-world classrooms. In addition, the sample consisted exclusively of adult learners, many of whom were university students. The extent to which the observed feedback dynamics generalize to younger learners or school-based educational contexts therefore remains an open question. Future studies should therefore examine how similar automated feedback mechanisms work over longer time periods, with more diverse learner populations, and across different types of learning tasks and contexts."*

<u>Reviewer #2 (Remarks to the Author):</u>

I appreciated reading this innovative paper. The study examines the effects of adaptive feedback on learning and takes individual learning characteristics into account. The nature of the study offers many starting points for further research, and the results are very interesting and relevant both on a theoretical level for real-time insights into the processing of feedback and on a practical level for the development of intelligent tutorial systems, which are becoming increasingly important in education. I think the manuscript is very interesting for the feedback community and a wilder field of AI in education.
The introduction and discussion are very well written and coherent, covering the important and relevant theoretical background and painting a coherent picture. There are still a few points that could be improved in the results section.
Below, I highlight specific sentences and sections with remarks and comments that need to be revised.

Thank you for the overall positive feedback. We appreciate the thoughtful comments and have carefully addressed each of them in detail below to enhance the clarity and presentation of our study, especially the results section.

- The last sentence in the abstract is rather unspecific. You might formulate a more meaningful sentence.

We have revised the final sentence of the abstract to make the contribution more specific and informative (lines 25-27). In addition, the abstract was revised more broadly to improve clarity and to align with the journal's requirements.

> Abstract, lines 25-27:

“These findings highlight that the effectiveness of automated failure feedback depends on both its design and learners’ situational cognitive and emotional states, illustrating how a situational, temporally sensitive approach can help open the “black box” of feedback effectiveness.”

- I was wondering if you have an ethical votum and a preregistration for this study.

Thank you for this question. Ethical approval for the study was obtained from the University of Potsdam Ethics Committee and is reported in the Methods section (line 217). The study was not preregistered; we have now added an explicit statement to this effect in the Methods section (lines 217-218).

Methods, lines 217-218:
Ethical approval for this study was granted by the University of Potsdam Ethics Committee (number 78/2023). The study was not preregistered.”

- In the section starting at line 147, I would appreciate it if a little more information were provided about the characteristics of the feedback in order to gain a better understanding, especially since the methods are only described at the very end of the paper and it is otherwise difficult to understand the results. (see also the next three points)

We understand that the description of the feedback characteristics may have been difficult to follow in the previous version of the manuscript. Following the editor’s request, the manuscript structure was revised so that the Methods section now appears directly after the Introduction, which should already improve readability and make the methodological details more accessible to the reader to understand the results. In addition, we clarified and restructured the description of the feedback characteristics in the Introduction (lines 160-185). The specific aspects raised in the following comments were addressed individually, as detailed below.

o The definition and the difference of fixed and adaptive frequency could be explained in more detail. Reading it for the first time, I was not sure what exactly is meant by this. Fixed feedback could be also associated with content as giving every time failure feedback regardless of the correctness of the answer. So, the word “fixed” can easily be associated with content. Maybe it would help to make this more explicit.

To address this comment, we revised the description of the experimental conditions to make more explicit that “fixed” and “adaptive” refer to the frequency of feedback delivery, not to its content (lines 164-166). The revised text now states that in condition 1, failure feedback was delivered after every incorrect response (fixed frequency), whereas in conditions 2 and 3, feedback frequency varied adaptively based on learners’ recent task performance and on-task enjoyment. It also clarifies separately that feedback content was generic in conditions 1 and 2, but personalized in condition 3.

Introduction, lines 164-166:
“In the fixed guidance condition (condition 1), failure feedback was delivered after every incorrect response (fixed frequency), resulting in a constant feedback frequency across incorrect responses (100%).”

o A few more information on how the feedback looks like would be helpful, especially what adaptive frequency means. What is the range - how often was adaptive feedback given?

We agree that the initial description of the feedback characteristics in the Introduction could be clearer. While the detailed implementation is described in the Methods section, we expanded the overview of the feedback design in the Introduction to more clearly explain how adaptive frequency was implemented. Specifically, we now clarify that feedback was delivered probabilistically, with the frequency ranging from 0% to 100% and being adjusted based on learners' prior block performance and self-reported enjoyment. These additions can be found in lines 166-174:

> Introduction, lines 166-174:
> *"In the basic-adaptive condition (condition 2), failure feedback was delivered adaptively across incorrect responses depending on learners' recent cognitive and emotional states (adaptive frequency; ranging from 0% to 100% based on learners' task performance and self-reported on-task enjoyment). In the personalized-adaptive condition (condition 3), the same adaptive frequency delivery strategy was applied (0% to 100%), but additionally the content of failure feedback was personalized to each learner's mistakes and previous steps in the system during learning task engagement (e.g., highlighting repeated errors or referencing earlier steps in the task; personalized content). Thus, the feedback content (generic or personalized feedback) and its presentation (fixed or adaptive frequency) were systematically varied between conditions."*

o It would also be helpful to add here if the tasks of the vocabulary learning game build on each other or are independent from each other.

Thank you for raising this helpful suggestion. We have clarified that the vocabulary learning task was not based on independent trials, but that learners gradually inferred word meanings through trial and error, allowing knowledge gained in earlier placements to inform later ones. This clarification has been added to both the Introduction (lines 176-179) and the Methods section (lines 240-241).

> Introduction, lines 176-179:
> *"Learners' received these failure feedback prompts after incorrect placements throughout the 30-minutes interactive learning session, during which they repeatedly placed objects in the room and gradually deduced the meanings of the instructions to find each objects correct position."*

> Methods, lines 240-241:
> *"Over the course of the task, learners progressively inferred the meanings of the Swahili instructions through trial and error, leading to knowledge gained from earlier placements informing later ones."*

- In the whole result section, the references for tables and figures are missing. Please insert them in every section at the respective places.

We have added explicit in-text references to the relevant tables and figures throughout the Results section to improve clarity and readability (e.g., lines 396, 397, 402, 405, 407, 415, 420, 428, etc).

- The lines 204-209 do not contain the results for H1. I would recommend placing this general section above line 203 to make the structure clearer.

Thank you for this suggestion. Following the revision of the manuscript structure, the Methods section now precedes the Results section, and the statistical model is already fully described there. To avoid duplication and improve clarity, we removed the paragraph about the model fit indices from the Results section.

- Please check the labels of the conditions in Table 3 and the result section, especially for H2 (ll. 214-233). The labels do not correspond exactly, which is a bit confusing.

We revised the labels of the conditions in Table 3 to ensure consistency and avoid confusion (see Table 3).

- I am not sure if the interpretation on the results for H2 in the discussion can really been explained by the cognitive load theory. There would need to be a big difference in the amount or the characteristics of feedback between the adaptive and the personalized-adaptive condition. Are there for example more words used for the personalized-adaptive feedback compared to the adaptive feedback?

We appreciate this thoughtful comment, as it raises an important point regarding the interpretation of one of our key findings. We agree that the applicability of cognitive load theory depends on whether the manipulation of the feedback plausibly affected the amount or structure of the information the learners needed to process. In the present study, this assumption is supported by the design, as in the personalized-adaptive condition the feedback messages contained more specific information by referencing learners' specific errors or prior steps, which indeed resulted in more words. As a result, these prompts were not only longer but also included more interacting elements that needed to be processed, compared to the more generic prompts used in the basic-adaptive condition (e.g., "*What made you choose this position? Try to reflect on your steps*" vs. "*What made you choose this position for *object*? Try to reflect on your steps – you could have known it wasn't correct*"). From a cognitive load theory perspective, such increases in informational specificity and element interactivity are theoretically expected to increase processing demands (Sweller, 1988), even if the absolute differences in length are moderate. This interpretation is consistent with prior research showing that increased sentence complexity in instructional materials can lead to higher mental effort during processing (DeLeeuw & Mayer, 2008). We have improved this explanation and included this citation to the manuscript (see lines 527-529). However, we agree that this mechanism cannot be directly tested with the current data and therefore only represents one possible interpretation rather than a definitive explanation. To address this concern, we revised the Discussion by (a) explicitly describing how the personalized feedback differed from the generic feedback in terms of informational complexity and sentence length (see lines 523-525; 528-531), and (b) broadening the interpretation of this finding by incorporating alternative theoretical explanations (see lines 522-554). These revisions position cognitive load theory as a theoretically grounded interpretation while avoiding overinterpretation of the findings.

> Discussion, lines 523-525:
> *"Personalizing the content of failure feedback resulted in prompts containing more specific information, which also increased sentence length and, consequently, the amount of information that needed to be processed."*
>
> Discussion, lines 529-532:
> *"In line with the cognitive theory of multimedia learning[43], this reflects the limited capacity of working memory, such that longer and more detailed feedback messages, like those used in*

the personalized-adaptive condition, may have required learners to process too many elements at once, thereby reducing immediate performance."

- As you mentioned in the sample description, that the no-feedback condition was not used for this paper, I was wondering if there are any other related papers, which use the no-feedback condition or if this study was part of a bigger project. If this is the case, please mention this in the method section and indicate any publications or pre-registrations relating to the paper.

The study reported here was part of a larger research project, and a related paper using the same dataset has previously been published (Ackermann et al., 2024). While the previous paper focused on overall effects of adaptive guidance at a more global level, the present manuscript takes a different perspective by examining the underlying mechanisms of how feedback operates during the learning process. Specifically, the current study adopts a fine-grained, situational approach by analyzing trial-by-trial dynamics, allowing us to capture how feedback effects unfold in real time and how they are shaped by cognitive and affective processes during task engagement. This provides a more detailed understanding of the processes through which feedback influences learning, complementing the more outcome-focused perspective of the earlier work. To ensure transparency, we have now added a statement in the Methods section describing the relation between the two publications and citing the earlier paper (see lines 256-258).

Methods, lines 256-258:
"A related paper using the same dataset examined general effects of adaptive guidance on learning outcomes, emotional experience, and self-regulated learning[32], whereas the present study focuses on trial-level mechanisms of feedback effectiveness."

- I am not sure what exactly happened in the adaptive frequency condition when no feedback is given. Was it indicated that the answer was correct or incorrect or did the participants directly answer the next question?

We appreciate this clarification question, which helped us to improve the study presentation in the Methods section. In all conditions, the robot consistently indicated whether a placement was correct or incorrect. The adaptive manipulation only determined whether an additional failure feedback prompt was provided after incorrect placements. If no such prompt was delivered, the interaction proceeded directly to the next trial following the correctness feedback. We have clarified this in the Study Design section (lines 234-240).

Methods, line 234-240:
"Regardless of condition, the robot always indicated whether a placement was correct or incorrect and the adaptive manipulation only determined whether an additional cognitive or metacognitive failure feedback prompt was provided additionally to the correctness feedback. After each placement, participants could freely choose the next object for the next trial. Correctly placed objects remained on their boxes, and the participant selected a new object. If a placement was incorrect, the object remained unsolved and could be attempted again in subsequent trials until it was correctly positioned."

- Please indicate how many datapoints were replaced by FIML (l. 581)?

No datapoints were replaced or imputed, as missing data were handled using Full Information Maximum Likelihood (FIML), which uses all available data during model estimation. We have clarified the nature and extent of missing data in the Methods section (lines 387-391). Specifically, missing values were structural, including missing prior block performance for first blocks (*n* = 733) and missing outcome values for the final incorrect trial of eight participants (*n* = 8). We have added the specific numbers to the Methods section as well.

> Methods, lines 387-391:
> *"Structural missing data (e.g., missing prior block performance for first blocks; n = 733 missing datapoints) were retained in the model and handled using Full Information Maximum Likelihood (FIML) under the MLR estimator. The outcome variable (next-trial correctness) was missing for the final incorrect trial of eight participants (n = 8; 0.36%) because no subsequent response existed. The model variables did not contain any missing data that was not structural."*

- In Table 1, if find the different values without labeling a bit confusing. Can you indicate which numbers represent cases and which numbers represent participants.

Thank you for pointing this out. In Table 1, the sample sizes differ because variables are reported at three hierarchical levels (placements, blocks, and learners). In addition, the number of placement- and block-level observations can vary slightly between conditions because participants differed in the number of trials and blocks completed within the fixed time window of the task. To improve clarity, we have revised the column names and the table note to explicitly indicate that N and n refer to the number of observations at the respective level (placements for Level 1, blocks for Level 2, and learners for Level 3; see lines 809-817).

- I was also wondering if mean and SD of task performance for Condition 1 and 2 are exactly the same in Table 1.

We double-checked the descriptive statistics to ensure that no error occurred in Table 1. The mean and standard deviation of task performance for Conditions 1 and 2 are identical in Table 1 because the values are reported rounded to two decimal places for clarity and consistency, following standard reporting conventions. In the raw data, the values differ slightly (Condition 1: *M* = 0.274683, *SD* = 0.446637; Condition 2: *M* = 0.273936, *SD* = 0.446273), which both round to the same values in the table.

- In Figure 1 the dashed lines are not clearly visible in a printed version. It might be useful to use bold dashed lines.

Thank you for this helpful suggestion. We have revised Figure 1 by increasing the line weight of the dashed lines to improve visibility in print. In addition, we adjusted the layout, font size, and overall design of the figure to enhance readability and align with the journal's formatting guidelines. For consistency, these formatting improvements were applied to all figures.

Reviewer #3 (Remarks to the Author):

Summary:
Testing adults, the authors were interested in determining how the type of feedback influenced learning for a technology based task. The authors state that while automated feedback for technology-based

learning environments is growing in popularity, the true effectiveness of these systems is not known due to variations in type of feedback used in previous studies and the lack of information available to assess how learners perceive feedback. To address these gaps in the literature, the authors analyzed a dataset from a previous experiment with a vocabulary learning game in which the function, content, and presentation of feedback from a robot were controlled. The first condition had "fixed" guidance for every incorrect response, the second condition was the "basic-adaptive" feedback condition which measured task performance and enjoyment, the third condition was the "personalized-adaptive" condition where both frequency and content were adaptive. Regardless of the conditions, half of the feedback prompts were cognitive and the other half were metacognitive. Fluent German speakers were recruited as participants and used the touch screen to move objects to a location. Objects and the location were presented to the participants in Swahili, feedback for incorrect trials (placements) was provided in German. A five-point Likert scale was used to capture self-reported emotion assessments (boredom and enjoyment) every 5 minutes. Their results showed that metacognitive feedback was significantly related with task performance (correcting a previously incorrect placement of an object), while cognitive feedback showed no correlation. They also found that learners with higher cognitive ability had better performance. Emotional assessments were not significantly correlated with block-aggregated task performance. A model testing the impact of feedback type on performance revealed that both cognitive and metacognitive feedback types had significant positive effects on performance. A separate model revealed that feedback frequency did not predict performance, so there was no difference in fixed vs basic-adaptive conditions. Another model revealed that even though both cognitive and metacognitive feedback types impacted performance, participants in the personalized-adaptive condition surprisingly benefitted less than basic-adaptive feedback for cognitive feedback: people with generic feedback performed better for cognitive feedback. Learners improved more with cognitive feedback in blocks with higher self-reported boredom while enjoyment was not significant. Learners with higher cognitive ability benefited less from cognitive feedback. The study results are interesting, and will be useful for people interested in measuring the effects of feedback, especially future studies involving imaging tools and data driven methods.

Overall Review: This manuscript is very applicable and presents many interesting questions about the quality of feedback in relation to performance. While the study is good, it would be greatly improved with some revisions, particularly by (1) adding clarification details for the methods that allow readers to replicate the study and (2) making the results as interpretable as possible for readers.

Thank you for the summary and positive overall assessment of the manuscript. We appreciate the recognition that the study addresses an important topic and raises relevant questions about feedback processes in technology-based learning. At the same time, the summary suggests that some aspects of the study design and analysis may not have been fully clear in the previous version of the manuscript. For example, the object placements were performed physically in the room rather than on a touch screen, and the analyses were conducted within a single three-level model rather than across multiple separate models. To improve clarity and interpretability, we carefully revised several sections of the manuscript to better explain the task procedure, experimental setup, and analytical approach. These clarifications are described in detail in the responses to the specific comments below.

Comments by Section:

Introduction

1. Line 48: please define meta-task processes

We have adapted the sentence as follows (line 55-57):

Introduction, line 55-57:
"According to this theory, feedback exerts its influence on learners' performance by changing their attentional focus: it may direct attention to the task, to the self, or to meta-task processes (i.e., higher-level processes above the task, such as self-related evaluation or affect)[7]."

2. Line 76: please provide an example of "metacognitive" functions of feedback. You do this in the methods, but it would be helpful for the readers to have some sense of a difference between cognitive and metacognitive feedback functions when you describe the procedure in Line 168. Also, please be consistent in how metacognitive is used, either "meta-cognitive" or "metacognitive".

Thank you for this comment. To make the distinction between cognitive and metacognitive feedback clearer earlier in the manuscript, we added a brief example of metacognitive feedback in the Introduction when describing the feedback functions (lines 81-86). In addition, we reviewed the manuscript to ensure consistent use of "metacognitive" throughout the text.

Introduction, lines 81-86:
"Feedback function concerns how the feedback influences student learning and may be either cognitive by supporting learning and performance through task-related information (e.g., providing information that helps learners process task-relevant material), motivational-affective by enhancing motivation and positive emotions (e.g., encouraging effort and persistence), or self-regulatory (metacognitive) by fostering learners' ability to plan, monitor, and evaluate their own learning (e.g., prompting learners to reflect on their performance or to evaluate their understanding)[12,13]."

Results
3. Line 693, Table 1: For the effect sizes, can you change the font style to bold for the row values based on whether or not the differences between factors (1 vs 2, 1 vs 3, and 2 vs 3) were statistically significant?

Thank you for this suggestion. The effect sizes reported in Table 1 are descriptive Cohen's d values and are not based on statistical hypothesis tests. Statistical significance was evaluated within the multilevel path model reported in Table 3. Because Table 1 is intended to present descriptive statistics rather than inferential comparisons, we did not highlight values based on statistical significance. To clarify this, we added a note to Table 1 indicating that the reported effect sizes are descriptive and not inferential tests (lines 816-817).

"Effect sizes represent descriptive differences between conditions and are not inferential significance tests."

4. Line 214 - 258: For the results mentioned in the "Moderating effects of feedback characteristics (H2)", "moderating effects of learner characteristics (H3)" and "additional findings: direct effects of moderators and control variables", are any of the results presented in the three tables or three figures? If so, can you include a reference to the appropriate table for each result in the text? For these sections, can you also consider including the mean differences and p values for the comparisons in the text directly?

We revised the Results section to include explicit references to the corresponding tables and figures throughout (see also response to comment 8 of Reviewer #2). In addition, we now report the relevant

model coefficients and p-values directly in the text for the main and moderating effects (e.g., lines 413-415).

> For example, Results, lines 413-415:
> *"Results indicate that both types of failure feedback, cognitive (B = 0.13, p = .01) and metacognitive (B = 0.18, p < .01), had significant and positive direct effects on task performance as compared to no feedback (see Table 3)."*

Discussion

5. Line 294 - 296: Did the authors consider a condition for a future study in which participants had control over feedback frequency? This would be less automatic, but users would have more control.

Thank you for this suggestion. In the present study, feedback frequency was controlled by the system to allow a direct comparison between predefined adaptive strategies across experimental conditions. Allowing learners to regulate feedback frequency themselves would introduce an additional source of variability that would make it more difficult to isolate the effects of the adaptive algorithm and would address a different research question. However, we agree that user-controlled feedback frequency represents an interesting alternative approach that could provide learners with greater autonomy. We have therefore added this as a direction for further research in the Discussion (see lines 634-637).

> Discussion, lines 634-637:
> *"In addition, it might be of interest to explore more learner-controlled forms of feedback delivery, for example by allowing learners to regulate the frequency of feedback themselves. Such approaches could provide insight into how externally adaptive and learner-controlled feedback mechanisms differentially affect engagement and performance."*

6. Line 331 - 335: The finding that people with higher cognitive ability benefited less from feedback is interesting. Did the authors consider how cognitive ability would interact with performance for a more difficult but similar vocabulary task? A future study could test how feedback, cognitive ability, and task difficulty interact with performance for advanced learners.

> Thank you for this helpful suggestion. In the present study, task difficulty was not systematically manipulated, as all participants completed the same vocabulary-learning task. We agree that examining how feedback effectiveness interacts with cognitive ability under varying task difficulty is an important direction for future research. We have therefore added this point to the Discussion (lines 653-656), highlighting the possibility to investigate how task difficulty may shape the relationship between cognitive ability and feedback effectiveness.
>
> Discussion, lines 653-656:
> *"In addition, systematically varying task difficulty could provide further insight into how cognitive load and learner characteristics jointly shape feedback effectiveness, for example by examining whether the observed interaction between cognitive ability and feedback differs in more difficult versions of the task."*

Methods

7. Line 441: Is it possible to show a visual representation of an example trial from the experiment as a figure in the manuscript?

Thank you for this helpful suggestion. Because the task was performed in a physical environment using real objects rather than a computer interface, a screenshot of a trial is not available. However, the sequence of actions within a trial is illustrated schematically in Figure 2. To clarify this, we revised the figure caption (lines 854-862) and the Methods section to explicitly indicate that Figure 2 represents the sequence of events within a typical trial (lines 233-234).

> Figure 2 caption, lines 854-862:
> *"Note. A learning session consisted of up to six five minutes (5-min) time blocks. Within each block, learners completed multiple trials (placements) of the vocabulary learning task. In each trial, the learner selected an object, listened to the robot's placement instruction, and positioned the item in the room. The robot then provided feedback on correctness and the current placement combination. If the placement was incorrect, additional failure feedback could be given, with frequency determined by the experimental condition: fixed (given after every incorrect attempt) or adaptive (given selectively based on individual learner's prior performance and most recent enjoyment rating). At the end of each 5-min block, participants rated their current emotional experience, which was used to determine the feedback strategy in the subsequent block. Trials continued until all 12 objects were correctly placed or 30 minutes had elapsed."*

> Methods, lines 233-234:
> *"see Figure 2 for a schematic illustration of the described sequence of events of a single trial."*

8. Since the results are based on data from a previous experiment, please include the month(s) and year(s) in which the original data from the experiment was collected. If possible, include a reference to the published data so readers can check if they are interested in reading about the previous study.

We have added the month and year of data collection to the Methods section in the revised manuscript (lines 206-207). In addition, we now cite the previous publication based on the same dataset and clarify how the present study differs in its focus (lines 256-258).

> Methods, lines 206-207:
> *"Participants were recruited as part of a larger experiment on adaptive guidance in a robot-supported learning task conducted between February and June 2024 in Germany."*

> Methods, lines 256-258:
> *"A related paper using the same dataset examined general effects of adaptive guidance on learning outcomes, emotional experience, and self-regulated learning[32], whereas the present study focuses on trial-level mechanisms of feedback effectiveness."*

9. Line 453: On a related note to the previous comment, I was left wondering how the technology-based vocabulary experiment was controlled? Did participants see both a computer monitor and the robot or was the robot an all-in-one machine with a touch screen display? What is the physical size, resolution and sampling rate (Hz) of the display screen used for testing? How far did participants sit away from the robot and or screen? Please list the name of the software that controlled the procedure and/or controlled the vocabulary game. Please clarify how participants made their response for the learning phase. Based on what was described in the manuscript, I am assuming participants used their fingers for a touch and drag response to move the object, but was wondering if participants had to use the mouse or keyboard for any responses. Readers may want to try to use the software/game in future studies and this detail will allow

for better replication. If these details are already stated in a different article that had the original data, then you can point the reader to that article instead.

Thank you for raising this point. The task in the present study was conducted in a physical learning environment rather than through a computer interface. Participants interacted with real objects placed in the room and followed spoken instructions provided by the robot. They responded by physically placing the objects at the instructed locations. Therefore, the task did not involve a computer monitor, touchscreen interface, mouse, or keyboard input. We realize that the previous description may have unintentionally suggested a screen-based interaction and have therefore clarified this aspect of the experimental setup throughout the manuscript (e.g., see lines 160-163, 221-222, 228-230). Technically, the experimental system was implemented in Python 3.10 using the NAOqi framework. The robot operated as an autonomous interactive system connected to a custom-built setup in which each target location was equipped with NFC readers controlled by Arduino Nano microcontrollers (C++), allowing real-time detection of object placements. The code for the experimental system is available as indicated in the Code Availability statement. We hope these clarifications improve the transparency of the setup.

> Introduction, lines 160-163:
> *"For this purpose, we conducted an experiment in which learners received automated failure feedback (i.e., feedback after incorrect responses) while working on a cognitive learning task that involved interpreting foreign-language instructions and physically placing objects in a room."*
>
> Methods, lines 221-222:
> *"The learning goal was to find the correct placements of objects in the experimental room"*
>
> Methods, lines 228-230:
> *"For each trial the learner first freely chose an object, then listened to the robot's instruction including the three-word goal placement in Swahili (object – preposition – location), and then physically place the object on one of the possible target locations in the room."*

10. Line 441: Is the reason for there being more locations (16) than objects (12) so that participants cannot rely on the process of elimination for the last few incorrect targets? If so, this detail should be included in the methods.

Thank you for pointing this out. Yes, the number of locations exceeded the number of objects to prevent participants from solving the remaining placements by simple process of elimination. We have added this clarification to the Methods section (lines 226-228).

> Methods, lines 226-228:
> *"The number of possible locations (16) exceeded the number of objects (12) to prevent participants from solving the remaining placements by simple process of elimination toward the end of the task."*

11. Line 449: "The learning phase ended when all twelve objects were correctly positioned or when 30 minutes had elapsed." This makes it sound like each participant completed only 12 trials. At Line 469, the manuscript says "after each five-minute block", which sounds like there were 12 trials per block that ended after all responses were corrected or after 5 minutes had elapsed. Did each participant complete

6 blocks? Either way, please update the sentence at line 449 so readers know how many trials and blocks were completed by each person in the entire experiment.

Thank you for pointing out this potential source of confusion. In the task, participants repeatedly attempted to place twelve objects until all were correctly positioned or until 30 minutes had elapsed. Thus, the total number of placements (trials) varied across participants depending on how quickly they solved the task. Emotional assessments were collected at five-minute intervals, resulting in up to six time blocks per participant. To clarify this structure, we revised the description of the task procedure in the Methods section (lines 237-244).

> Methods, lines 237-244:
> *"After each placement, participants could freely choose the next object for the next trial. Correctly placed objects remained on their boxes, and the participant selected a new object. If a placement was incorrect, the object remained unsolved and could be attempted again in subsequent trials until it was correctly positioned. Over the course of the task, learners progressively inferred the meanings of the Swahili instructions through trial and error, leading to knowledge gained from earlier placements informing later ones. As participants attempted to place the twelve objects on their respective correct location through repeated trials, the total number of trials varied between participants depending on how quickly they solved the task. The learning phase ended when all twelve objects were correctly positioned or latest when 30 minutes had elapsed."*

12. Line 469-470: The "pre-defined matrix to select one out of six possible probability levels" is a good way to gauge performance and emotional assessment across time. Readers might be interested in knowing the distribution of each probability level for the adaptive conditions. Can you report the percentage of blocks in which participants had a probability level of 0, percentage of blocks when participants had a probability of 20, 40, 60, 80, and 100%? This would be useful for highlighting task difficulty and learning between blocks. You could also look at block order: based on your hypotheses, you might expect to see increased probabilities (80% or higher) for Block 1 due to low performance and lower probabilities (20% or lower) for Block 6 due to better performance and learning for the task. This would be similar to the trial error statistics mentioned in lines 509 - 510 except applied to the probability levels for the adaptive conditions.

Thank you for this helpful suggestion. To provide more transparency about how the adaptive algorithm operated over time, we examined the distribution of feedback probability levels across blocks in the two adaptive conditions and now report these descriptively in the Supplementary Information (Supplementary Table S1 and Supplementary Figure S1). The supplementary material shows the full percentage distribution of the six probability levels (0%, 20%, 40%, 60%, 80%, 100%) for each block separately and for each condition. Across the adaptive conditions, higher probability levels were more frequent in earlier blocks, whereas a broader range of lower and intermediate probability levels appeared in later blocks. At the same time, the number of participants decreased across blocks because some learners completed the task earlier and did not continue to later blocks. Therefore, the distributions in later blocks should be interpreted with caution, as they reflect only the subset of participants who had not yet completed the task. Because these distributions are descriptive and not directly related to the main hypotheses, we did not integrate the full table and figure into the main text. Instead, we added a short reference to the supplementary material in the Methods section.

> Methods, lines 273-274:

“The resulting probability levels are descriptively provided in the Supplementary Information (Table S1 and Figure S1).”

Supplementary Information, Table S1:

Table S1. *Distribution of failure feedback probability levels across blocks and conditions.*

Variable	Condition 1 *Fixed Guidance Condition*		Condition 2 *Basic-Adaptive Condition*		Condition 3 *Personalized-Adaptive Condition*	
	n	*%*	*n*	*%*	*n*	*%*
**Block 1 (minutes 1-5)**	**30**	–	**30**	–	**30**	–
Probability level 0%	30	100.0	30	100.0	30	100.0
Probability level 20%	0	0.0	0	0.0	0	0.0
Probability level 40%	0	0.0	0	0.0	0	0.0
Probability level 60%	0	0.0	0	0.0	0	0.0
Probability level 80%	0	0.0	0	0.0	0	0.0
Probability level 100%	0	0.0	0	0.0	0	0.0
**Block 2 (minutes 6-10)**	**30**	–	**30**	–	**30**	–
Probability level 0%	0	0.0	0	0.0	3	10.0
Probability level 20%	0	0.0	0	0.0	0	0.0
Probability level 40%	0	0.0	0	0.0	0	0.0
Probability level 60%	0	0.0	12	40.0	11	36.7
Probability level 80%	0	0.0	12	40.0	12	40.0
Probability level 100%	30	100.0	6	20.0	4	13.3
**Block 3 (minutes 11-15)**	**30**	–	**30**	–	**30**	–
Probability level 0%	0	0.0	5	16.7	1	3.3
Probability level 20%	0	0.0	1	3.3	4	13.3
Probability level 40%	0	0.0	0	0.0	0	0.0
Probability level 60%	0	0.0	10	33.3	8	26.7
Probability level 80%	0	0.0	8	26.7	10	33.3
Probability level 100%	30	100.0	6	20.0	7	23.3
**Block 4 (minutes 16-20)**	**26**	–	**22**	–	**25**	–
Probability level 0%	0	0.0	1	4.5	2	8.0
Probability level 20%	0	0.0	1	4.5	3	12.0
Probability level 40%	0	0.0	2	9.1	0	0.0
Probability level 60%	0	0.0	8	26.4	2	8.0
Probability level 80%	0	0.0	5	22.7	9	36.0
Probability level 100%	26	100.0	5	22.7	9	36.0
**Block 5 (minutes 21-25)**	**20**	–	**16**	–	**16**	–
Probability level 0%	0	0.0	2	12.5	1	6.2
Probability level 20%	0	0.0	0	0.0	5	31.2
Probability level 40%	0	0.0	0	0.0	0	0.0
Probability level 60%	0	0.0	5	31.2	2	12.5
Probability level 80%	0	0.0	5	31.2	4	25.0
Probability level 100%	20	100.0	4	25.0	4	25.0
**Block 6 (minutes 26-30)**	**12**	–	**12**	–	**11**	–
Probability level 0%	0	0.0	1	8.3	0	0.0
Probability level 20%	0	0.0	1	8.3	2	18.2
Probability level 40%	0	0.0	0	0.0	1	9.1
Probability level 60%	0	0.0	3	25.0	2	18.2
Probability level 80%	0	0.0	2	16.7	5	45.5
Probability level 100%	12	100.0	5	41.7	1	9.1

Note. Values show counts (*n*) and percentages (*%*) of participants per block and condition at each feedback probability level. Bold *n* values reflect participants contributing data in that block; decreases across later blocks indicate task completion. Percentages are calculated relative to the number of participants contributing data in each block.

Supplementary Information, Figure S1:

Figure S1. *Distribution of failure feedback probability levels across blocks and conditions.*

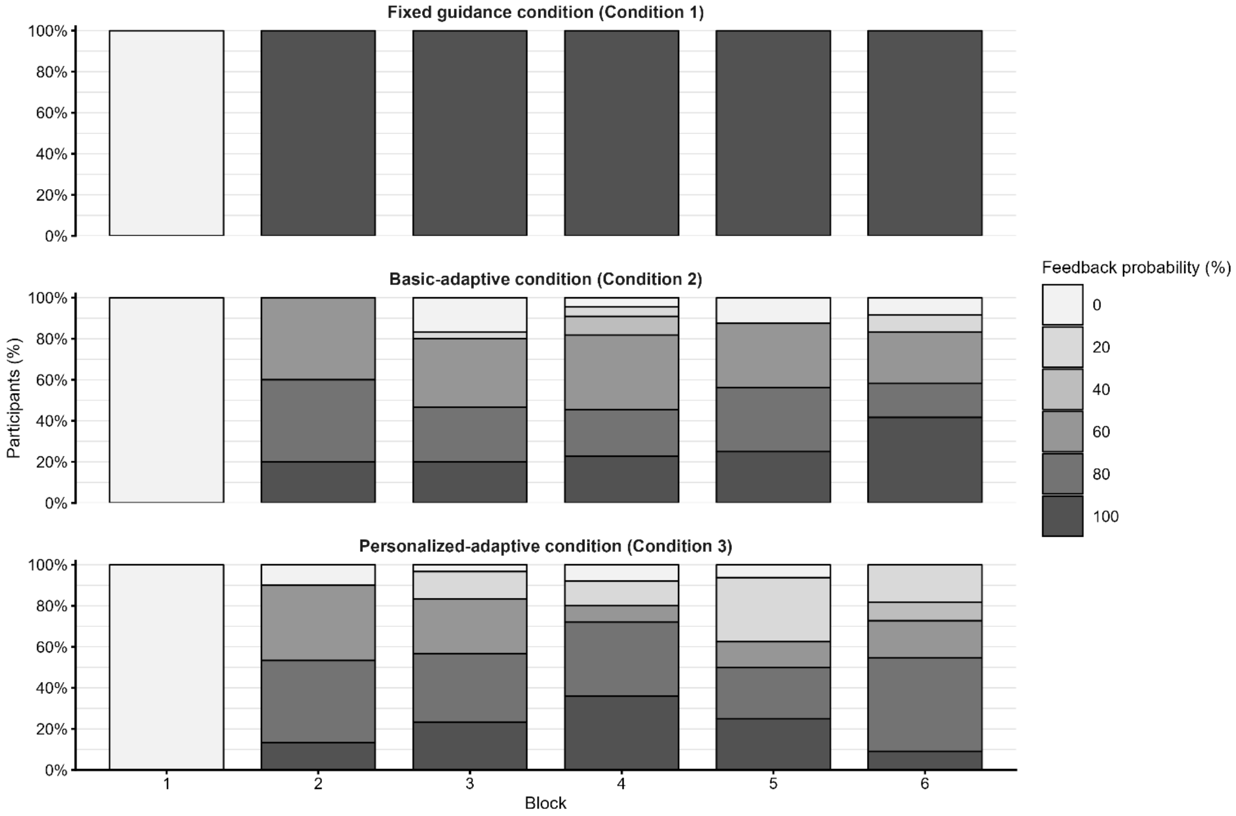

Note. Stacked bar charts show the percentage of participants assigned to each feedback probability level (0%, 20%, 40%, 60%, 80%, 100%) within each block.

Other Comments

13. The study design section says that participants "listened to the robot's instruction" and that the "robot announced" the feedback in German. Given that the authors were interested in testing how different types of feedback influenced performance, did the authors consider how audio feedback would differ from visual feedback? Such as presenting the feedback as text on the touch screen instead of audio, or both. Or testing different types of the robot's facial expressions. These ideas could be included along with the suggestions in line 410 - 412.

Thank you for this valuable suggestion. In the present study, feedback was intentionally delivered through the robot's spoken instructions to maintain a natural social interaction format and to keep the feedback modality constant across conditions to isolate the effects of the feedback characteristics themselves. Introducing and varying additional feedback modalities, such as visual text or robot facial expressions, would have introduced an additional experimental factor that could confound the interpretation of the feedback effects. Prior work investigating the role of tutor embodiment in digital learning environments found that design features such as physical embodiment and interaction modality can influence learners' affective responses (e.g., enjoyment) independently of learning outcomes (Ackermann et al., 2025). Therefore, in the current study we intentionally kept the feedback modality constant to focus specifically on the impact of feedback characteristics. We nevertheless agree that systematically comparing different

feedback modalities is an important direction for future research and have added a corresponding note in the Discussion (lines 667-670).

> Discussion, lines 667-670:
> *"In addition, future studies could systematically examine how the modality of feedback presentation (e.g., auditory, visual, or multimodal combinations of speech, text, and expressive cues) influences learners' engagement and processing of feedback, as prior research suggests that such design features can influence learning outcomes[55].*

Reviewer #1 (Remarks to the Author):

I thank the authors for their thoughtful and thorough response to my previous comments. The revised manuscript shows substantial improvement in clarity, theoretical framing, and methodological transparency. In particular, the clearer distinction between findings that confirm existing theories and those that extend them effectively addresses my earlier concern. Before final acceptance, I have one remaining point that I believe would further strengthen the manuscript.

The authors now acknowledge that no manipulation check was conducted to verify whether learners perceived cognitive and metacognitive feedback as functionally distinct. This is an honest and appropriate addition. I would encourage the authors to go one small step further by noting how this limitation constrains the interpretation of the differential effects observed, for instance, the finding that boredom moderated only the effect of cognitive feedback. Readers would benefit from seeing this caveat linked explicitly to the corresponding findings in the Discussion rather than appearing only in the general limitations paragraph.

We agree that the limitation regarding the absence of a manipulation check could be linked more directly to the corresponding findings in the Discussion. We therefore revised the section discussing the moderating effect of boredom to explicitly state that conclusions regarding differential effects between cognitive and metacognitive feedback should be drawn cautiously, as the present study did not assess whether learners perceived these feedback types as functionally distinct during the task. These changes can be found in lines 587-591:

> *"At the same time, the differential effects observed for cognitive and metacognitive feedback should be interpreted cautiously, as the present study did not assess whether learners perceived these feedback types as functionally distinct during the task. Although the prompts were theoretically categorized, it remains unclear to what extent learners subjectively differentiated between them during task engagement."*

Reviewer #2 (Remarks to the Author):

Dear Authors,
thank you for your thorough response to my comments and questions. I am overall satisfied with you editing. I have just one more comment.
Regarding the changes you made in the discussion for the interpretation with cognitive load theory. I appreciate the revised interpretation. However, I feel that the mention of CTML here is redundant and inappropriate. CTML is based on cognitive load theory, and the argument put forward is exactly the same. Furthermore, CTML does not align with the feedback characteristics, as your study involves text- / speech-based feedback only and does not use additional materials such as pictures. Please revise this section again.

Thank you for pointing this out. As the present study did not involve multimedia instructional materials, the reference to the cognitive theory of multimedia learning (CTML) could indeed distract from the main argument. Our intention was to refer more generally to the limited capacity of working memory during information processing, which is already sufficiently covered by cognitive load theory itself. We therefore removed the CTML reference and revised the paragraph. The changes can be found in lines 531-533:

> *"From a cognitive load perspective, this reflects the limited capacity of working memory, such that longer and more detailed feedback messages, like those used in the personalized-adaptive condition, may have required learners to process more elements simultaneously, thereby reducing immediate performance."*

<u>Reviewer #3 (Remarks to the Author):</u>

I have no further comments. The manuscript should be accepted.
